# Supplementary material for: Systematic shifts in scaling behavior based on organizational strategy in universities
Source: PLoS One. 2021 Oct 28;16(10):e0254582. doi: 10.1371/journal.pone.0254582 (PMC8553050; doi:10.1371/journal.pone.0254582)
Supplement: S1 File — (PDF) [file pone.0254582.s001.pdf]

SUPPLEMENTARY MATERIALS

to

SYSTEMATIC SHIFTS IN SCALING BEHAVIOR BASED ON ORGANIZATIONAL STRATEGY IN UNIVERSITIES

Ryan C. Taylor[1,3], Xiaofan Liang[2],
Manfred D. Laubichler[3,4], Geoffrey B. West[5], Chris P. Kempes[5], Marion Dumas[6]
1 – School of Sustainability, Arizona State University; 2 – Minerva University; 3 – Global Biosocial Complexity Initiative, Arizona State University; 4 – School of Life Sciences, Arizona State University; 5 – Santa Fe Institute; 6 – Grantham Research Institute, London School of Economics and Political Science

TABLE OF CONTENTS

Appendix A: Data documentation 4
Appendix B: Precise definitions for key variables used 6
Appendix C: Total enrollment headcount as the underlying size variable 14
Appendix D: University sector definitions and outlier analysis 16
Appendix E: Delta Cost Project grouping of campuses and institutions 29
Appendix F: Revenue and expenditure component analyses 38
Appendix G: Completion rate analyses 45
Appendix H: Tuition, selectivity and post-graduation earnings 50
Appendix I: Overall magnitude analysis of higher education 51
Appendix J: Final table of all scaling results 60
Appendix K: References 63

TABLES

Table B-1 8
Table C-1 15
Table D-1 17
Table D-2 18
Table D-3 24
Table D-4 29
Table E-1 30
Table E-2 32
Table E-3 36
Table E-4 36
Table F-1 45
Table G-1 46
Table G-2 47
Table G-3 49
Table H-1 51
Table J-1 61

FIGURES

Figure C-1 14
Figure D-1 17
Figure D-2 20
Figure D-3 21
Figure D-4 22
Figure D-5 22
Figure D-6 23
Figure D-7 25
Figure D-8 26
Figure D-9 27
Figure D-10 28
Figure D-11 28
Figure E-1 33
Figure E-2 34
Figure F-1 39
Figure F-2 40
Figure F-3 41
Figure F-4 42
Figure F-5 44
Figure F-6 44
Figure G-1 48
Figure G-2 49
Figure H-1 50
Figure H-2 51
Figure I-1 52
Figure I-2 53

Figure I-3 53
Figure I-4 54
Figure I-5 54
Figure I-6 55
Figure I-7 55
Figure I-8 56
Figure I-9 56
Figure I-10 57
Figure I-11 57
Figure I-12 58
Figure I-13 59
Figure I-14 60

Appendix A: Data documentation

Original data sources

For this paper, we conducted analysis on a combined dataset composed of matched data from the Delta Cost Project, based on Integrated Postsecondary Education Data System (IPEDS), with additional variables of completion metrics, selectivity and mid-career education returns from College Scorecard Project, Brookings Value-Added Dataset and the Equality of Opportunity Project respectively.

IPEDS is the main postsecondary data collection program of the National Center for Education Statistics (NCES). It administers mandatory surveys to all postsecondary educational institutions in the United States and its territories that have a Program Participation Agreements (PPA) with the appropriate office at the U.S. Department of Education. This PPA makes the institution eligible to accept Federal Student Aid-receiving students, as authorized by the Title IV of the Higher Education Act of 1965 (Ginder, Kelly-Reid and Mann 2014, Higher Education Act 1965). Therefore, universities that opt out from receiving federal funding for various reasons are not included in our analysis. However, for the purposes of this paper, the general terms "universities" and "schools" refer to these Title-IV postsecondary schools. The Higher Education Act of 1965 more specifically defines these Title-IV institutions as degree-granting institutions that admit as regular students only persons with a high school diploma or equivalent, are legally authorized by a state, and are accredited by an agency recognized by the U.S. Secretary of Education.

We primarily use 2013 data from the Delta Cost Project (www.deltacostproject.org), founded in 2007 by an independent non-profit of the same name. This dataset, which we abbreviate to "Delta data" or "Delta," brings together selected variables from the IPEDS surveys—most notably Institutional Characteristics, Fall Enrollment, Finance, Fall Staff and Graduation Rates—in order to facilitate in-depth comparisons between universities in the IPEDS universe (Hurlburt, Peek and Sun 2017). The Delta Cost Project standardizes data that had been imputed differently between reporting standards and years, as institutional groupings and the surveys themselves change over time (Hurlburt, Peek and Sun 2017, see also Appendix E). Key variables we used in the analysis include enrollment, faculty, revenues and expenditures; see Appendix B for a full list.

We supplement Delta data with data from the College Scorecard project, here "Scorecard," (U.S. Department of Education 2015, 2018, https://collegescorecard.ed.gov/), in order to examine student completion outcomes. The College Scorecard database was assembled primarily to help students and families assess college options. Scorecard integrates IPEDS metrics with National Student Loan Data System (NSLDS) data on completion, earnings and loan repayment for cohorts of Federal Student Aid-receiving (FSA) students, who are tracked via the Free Application for Federal Student Aid (FAFSA), the application for all federal student aid programs. The Delta Cost Project dataset also contains graduation rate data, for full-time, first-time degree-seeking students. Scorecard outcomes are more complete because they follow cohorts of FSA students, as discussed in Appendix G.

We acknowledge here that IPEDS has recently added the Outcome Measures (OM) Survey, which includes 2015 and 2016 graduation rate data for 6yr and 8yr cohorts of not only full-time, first-time students, but all undergraduates by full- / part-time and first- / non-first-time (transfer). This complete coverage of all undergraduate students is lacking in the outcome measures we use, namely FSA completions, and would circumvent many of the problems and assumptions outlined in Appendix G. However, one advantage of examining FSA completions, over OM, is that FSA attributes transfer student completions to their original institution, whereas OM does not distinguish between transferring away and dropping out. In the community college sector, where transferring is often considered a desirable outcome, using OM would make success rates appear artificially low. Despite exceeding the current scope, IPEDS Outcome Measure data may merit future investigation.

Post-graduation student performance metrics, such as mean earnings after graduation, all come from the Mobility Reports Cards by Chetty, Friedman, Saez, Turner, and Yagan (2017), as part of the Opportunity Insights Project, downloadable from https://opportunityinsights.org/data/mrc_table3.csv. This data provides the average earnings in 2014 of students having attended a particular institution for at least a year (whether they graduated or not), for cohorts born from 1980 to 1991. We use the data for the cohort born in 1984. This dataset is the best existing estimate of earnings as the data was obtained from matching record of student attendance with tax filings.

Two more datasets are used as complements. First, the Brookings Added Value dataset is used as it contains data on normalized SAT entrance scores (on the quantitative section of the SAT) for 3626 institutions. Finally, we also make use of the Higher Education R&D Survey (HERD) sponsored by the NSF. It requests institutions of higher learning granting a Bachelor or higher degrees to report the source of their R&D funding on a yearly basis. This dataset for 2013 includes data for 886 universities for which we have a valid IPEDS UNITID. We include the reported revenue (other than own institutional fund) as a robustness check for the Delta data, especially given that Delta data do not provide a direct measure of research revenue (we used the revenue component “Government Grants and Contracts” as a proxy for research revenue).

Merging the datasets

To merge College Scorecard and Brookings to Delta data, we begin with all 5,804 entries in Delta data that have values for total enrollment and remain after our outlier analysis described in Appendix D; we then search the pool of 7,804 Scorecard entries for entries with matching IPEDS UNITIDs. We append matches from Scorecard to Delta to create the combined dataset used in our analysis.

On one hand, there are 238 universities in Delta without matching UNITIDs in Scorecard that have valid enrollment data, which enrolled a total of 197,719 students. These Delta-only observations include the U.S. military academies, a few small research universities, and (mainly) 2yr and 4yr for-profit institutions. A list accompanies the other data files used in our analysis. The main example is 26 campuses of Devry University, which enrolled over 74,000 students combined. The problem arises from a Scorecard grouping together campuses or institutions that report earnings and other data for FSA receiving students together. Delta data does not include

such data and separates these schools by campus. We return to this problem in Appendix G on completion rate analyses.

On the other hand, there are 1,508 entries from Scorecard with UNITIDs that did not appear in Delta; however, 688 of these observations, accounting for 1,696,272 undergraduate students (neither total enrollment nor graduate enrollment are included in College Scorecard), actually find their way into Delta data as "child institutions," grouped under "parent institutions," and of these, 441 institutions with 405,075 students have usable FSA completions data. The remaining 820 Scorecard schools, that neither matched a Delta data entry nor a child institution wrapped into one, account for only 143,179 students. Appendix E explains in detail how Delta groups together campuses or institutions with common IPEDS reporting history while Scorecard disaggregates them into multiple institutions, and it addresses how this grouping affects our analysis. We conclude that campus and institutional grouping does not affect our main findings.

Figure 4 in the main text results from combining tuition data from Scorecard, data on incoming student test scores from Brookings, and data on earnings from the Equality of Opportunity project. The unit of analysis for the Mobility data is the super-OPEID, derived from the Office of Postsecondary Education Identifiers, but which aggregates some OPEID6 units into super-OPEID because multiple OPEID6 units were found to match the EIN-ZIP numbers indexing colleges in the 1098-T forms used as one of the primary datasets by Chetty et al. (2017). Hence, as we merge the data on average earnings with the tuition data from Scorecard and the test data from Brookings, we face a downscaling problem: for OPEID6 units that are part of a super-OPEID, we have to calculate the average for the super-OPEID that each institution is part of. The Equality of Opportunity dataset has data on earnings for the cohort born in 1984 for 2,756 schools. 2,482 of these could be matched to Scorecard using OPEID6 values, as well as a fuzzy string matching approach for the name of the institution (because Scorecard seems to have some false attribution of OPEID6 values). Overall, we have earnings data for 80% of public institutions, 58% of private non-profit institutions and 38%% of private for-profit institutions. The schools for which data is missing tend to be smaller (less than 5,000 students). Overall, 2,183 schools have data for all of the three following variables: earnings, standardized incoming test scores, and tuition.

Appendix B: Precise definitions for key variables used

Table B-1 includes detailed descriptions of each variable used in the analysis. They fall into seven categories: enrollment, revenues, expenditures, faculty, student outcomes, student earnings, and institutional characteristics. All revenue and expenditure data originally comes from the IPEDS Finance Survey through three state-level reporting standards: Governmental Accounting Standards Board (GASB) Statements 34 and 35 for most public institutions, (2) Financial Accounting Standards Board (FASB) for private and some public institutions (IPEDS 2014), or (3) FASB for for-profit institutions. All revenue and expense categories used here are reported consistently between the three standards, relevant exceptions noted below. See Hanover Research report (2014), Goldstein and Menditto (2017), and this IPEDS webpage: https://nces.ed.gov/ipeds/report-your-data/data-tip-sheet-distinguishing-finance-standards-fasb-gasb

The “Data Source” column provides reference to the dictionaries, glossaries and other documents where the original variable definitions are explained. For readability we condensed, rearranged or rewrote each definition. Where we merged variables, or the Delta Cost Project merged IPEDS variables, it is always explained in the first sentence of the definition.

Delta = Delta Cost Project dataset. Variable dictionary, data mapping file from IPEDS to Delta, and data file documentation (Hurlburt, Peek and Sun 2015) are all available here:
https://www.deltacostproject.org/delta-cost-project-database

IPEDS = Integrated Postsecondary Education Data System, descriptions of IPEDS surveys is available in their methodology report (Ginder, Kelly-Reid, and Mann 2014):
https://nces.ed.gov/pubs2014/2014067.pdf
A glossary of all terms in all IPEDS surveys is linked here:
https://surveys.nces.ed.gov/ipeds/Downloads/Forms/IPEDSGlossary.pdf
IC = Institutional Characteristics survey

SC = College Scorecard dataset. See Scorecard dictionary (n.d.), and for additional information see Scorecard documentation U.S. Department of Education (2018) or the presidential report (2015) on using Scorecard data:
https://collegescorecard.ed.gov/assets/CollegeScorecardDataDictionary.xlsx
https://collegescorecard.ed.gov/assets/FullDataDocumentation.pdf
https://collegescorecard.ed.gov/assets/UsingFederalDataToMeasureAndImprovePerformance.pdf
NSLCS = National Student Loan Data System
FAFSA = Free Application for Federal Student Aid, the application for all federal student aid programs and means of collecting data on FSA-receiving cohorts.

All years refer to data year, unless otherwise noted: for example, 2013 refers to the period from July 1, 2013 - June 30, 2014 (and September 1, 2013 – August 31, 2014 for schools not using a typical semester, trimester or quarter calendar system).

HERD = Higher Education Research & Development dataset. For more information, visit:
https://ncsesdata.nsf.gov/herd/2013/#tabs-2
NSF = National Science Foundation

MRC = Mobility Report Cards dataset
https://opportunityinsights.org/paper/mobilityreportcards/

Table B-1

Variable Name	Data Source	Description	Use
Total enrollment	IPEDS, Enroll-ment Fall	The total headcount of all students enrolled in the fall, taking courses creditable toward a degree or formal award. Equals the sum of four enrollment components used here:<br>1. full-time undergraduates<br>2. part-time undergraduates<br>3. full-time graduates<br>4. part-time graduates<br>Graduate students include both doctoral and masters-level students.	Underlying size variable for all revenue, expenditure, and faculty scaling analyses
Full-time equivalent (FTE) enrollment	IPEDS, Enroll-ment Fall	A measure of enrollment adjusted to consider intensity of enrollment, estimated by multiplying part-time enrollment by factors that vary by control and level of institution and level of student, and adding this estimated full-time equivalent amount to the full-time enrollment of the institution. This formula is used by the U.S. Department of Education to produce the full-time equivalent enrollment data published annually in the Digest of Education Statistics (NCES 2016). Typical measure of enrollment, used by education researchers (Presidential Report 2015, NCES 2016, Webber and Ehrenberg 2010).	Alternative size variable for all revenue, expenditure, and faculty scaling analyses, see Appendix C
Total revenue	IPEDS, Finance, Delta	The sum of all 2012-13 fiscal year revenue categories: student tuition, government student aid, government appropriations, government grants and contracts, donation and investment revenue, and auxiliary revenue. Institutional grant aid is not included.	Total revenue
Student tuition	IPEDS, Finance, Delta	The net amount of tuition revenue paid directly by students (not including Pell, Federal, State, and Local grants), whether from savings, Federal Student Loans, or other loan programs. Third party grants and scholarships and Veterans Affairs education benefits under the Post-9/11 or Montgomery GI Bill are also included. This amount approximates the total cost of education to students and families.	Component of revenue
Government student aid	IPEDS, Finance, Delta	Total student aid grant amounts, calculated as the sum of scholarships and fellowships from local government, those from state government, and the gross amount of federal educational grant aid disbursed or otherwise made available to recipients by the institution. Pell grants and all other grants issued by federal agencies are included, except Veterans Affairs education benefits. Loans and federal work study are also excluded.	Component of revenue

Government appropria-tions	IPEDS, Finance, Delta	The sum of local, state and federal appropriations. Below the state level, local appropriations consist of education district taxes assessed directly by an institution or on behalf of an institution, received immediately in full amount, and similar revenues from local policy based on collections of other taxes or resources (sales taxes, gambling taxes, etc.). State and federal appropriations are revenues received by the institution through acts of their respective legislative bodies. Funds reported in this category are for meeting current operating expenses, not for specific projects or programs. (In the delta data dictionary, federal appropriations are grouped with federal grants and contracts as restricted revenue, unlike state and local appropriations. However, the precise functions of these federal monies are not specified, so we assume they are more similar to state and local appropriations than government grants and contracts.) As such, grants, contracts and capital appropriations are excluded at all levels.	Component of revenue
Government grants and contracts, or Research revenue	IPEDS, Finance, Delta	The sum of local, state and federal grants and contracts. These revenues are reserved for research, training programs, or public service activities for which expenditures are reimbursable under the terms of a grant or contract issued by a government agency at any level (such as the National Science Foundation or California Energy Commission). Pell Grants are excluded if they were reported as federal grants. We use this variable as a proxy for research revenue.	Component of revenue
Donation investment revenue	IPEDS, Finance, Delta	The total amount of revenue coming from private gifts, grants and contracts, affiliated entities, and investment returns. Gifts are private donations involving no legal consideration, while private grants and contracts stipulate provision of specific goods and services to the funder for receipt of the funds. They directly relate to non-auxiliary institutional purposes, and include estimated dollar amount of contributed services. Affiliated entities include fundraising foundations, booster clubs, and other non-consolidated institutionally-related organizations. Investment income encompasses interest, dividend, rental and royalty income revenues derived from the institution's investments, especially those of endowment funds. (Endowment investment income may take the form of, and includes, both realized and unrealized gains and losses at FASB institutions, but not GASB institutions, which may explain some but not all of the gap between the endowments of public and private research universities.)	Component of revenue

Auxiliary revenue	IPEDS, Finance, Delta	The total amount of revenue from all operations of auxiliary enterprises, hospitals, independent enterprises, and other sources. Auxiliary enterprises are essentially self-supporting fee-based operations of the institution that exist to furnish a service to students, faculty, or staff, including residence halls, food services, student health services, intercollegiate athletics (only if essentially self-supporting), college unions, college stores, faculty and staff parking, and faculty housing. Independent enterprises are generally limited to major federally funded research and development centers, though expenses managed as investments of endowment funds are excluded. See delta variable dictionary for full description of miscellaneous / other revenues.	Component of revenue
Total expenditure	IPEDS, Finance, Delta	The sum of all 2012-13 fiscal year expense categories: instructional, research, public service, maintenance, student service, and grants and auxiliary expenditures. Institutional grant aid, depreciation, and interest payments are not included.	Total expenditure
Instructional expenditure	IPEDS, Finance, Delta	Expenses of the colleges, schools, departments, and other instructional divisions of the institution, including general academic instruction, occupational and vocational instruction, community education, preparatory and adult basic education, special and extension sessions (all of which are both credit and non-credit). This category also includes expenses for departmental research and public service that are not separately budgeted.	Component of expenditure
Research expenditure	IPEDS, Finance, Delta	Expenses for activities specifically organized to produce research outcomes and commissioned by an agency either external to the institution or separately budgeted by an organizational unit within the institution. The category includes institutes and research centers, and individual and project research.	Component of expenditure
Public service expenditure	IPEDS, Finance, Delta	Public service activities primarily provide non-instructional services beneficial to individuals and groups external to the institution, such as conferences, institutes, general advisory service, reference bureaus, community services, cooperative extension services, and public broadcasting services.	Component of expenditure
Maintenance expenditure	IPEDS, Finance, Delta	The sum of expenditures on academic support, institutional support, and operation and maintenance activities. Academic support expenditures support the primary institutional missions identified by IPEDS: instruction, research, and public service. Relevant services and activities include the retention, preservation, and display of educational materials (for example, libraries, museums, and galleries), academic administration (including academic deans but not department chairpersons), course and curriculum development expenses, formally organized academic personnel	Component of expenditure

		development, and services like information technology. Institutional support activities are typically understood as administration or bureaucracy. They include expenses for general administrative services, central executive-level activities concerned with management and long range planning, legal and fiscal operations, space management, employee personnel and records, logistical services such as purchasing and printing, and public relations and development. Operation and maintenance activities service campus grounds and facilities used for general, non-auxiliary purposes, with expenses including utilities, fire protection and property insurance. (GASB standards separate out operation and maintenance costs, but FASB standards embed them in each functional expense category, such instruction, research and student services. The Delta Cost Project harmonized all universities by subtracting operation and maintenance costs from all categories, and recombining them separately.) See Delta Documentation (Hurlburt, Peek and Sun 2017).	
Student service expenditure	IPEDS, Finance, Delta	Student service includes admissions, registrar activities, and activities whose primary purpose is to contribute to students emotional and physical well-being and to their intellectual, cultural, and social development outside the context of the formal instructional program. Examples include student activities, cultural events, student newspapers, intramural athletics, student organizations, supplemental instruction outside the normal administration, and student records. Intercollegiate athletics and student health services may also be included except when operated as self-supporting auxiliary enterprises.	Component of expenditure
Grants and auxiliary expenditure	IPEDS, Finance, Delta	The sum of grants to third parties and the operating expenses all auxiliary enterprises, independent enterprises, and hospitals that are reported as part of the university. Grants include scholarships and fellowships, paid to students or third parties for goods and services not provided by the institution, such as off-campus housing. These third party grants represent all financial aid counted towards total expenditures, since aid packages that students direct towards tuition and fees are not counted. See auxiliary revenue for a definition of auxiliary and independent enterprises.	Component of expenditure
Total faculty	IPEDS, Fall Staff, Delta	The total number of persons identified by the university in 2013 whose initial assignments principally entail conducting instruction, research or public service. They may hold academic rank titles of professor, associate professor, assistant professor, instructor, lecturer, an equivalent, or even executive titles (e.g. dean,	Potential mechanisms for observed

		department head, provost) *if* their principal activity is instruction combined with research and/or public service. Calculated as the sum of four faculty components listed here:<br>1. Full-time faculty<br>2. Part-time faculty<br>3. Teaching graduate students<br>4. Non-teaching graduate students<br>All counts include both medical and non-medical personnel.	educational scaling
Faculty pay	IPEDS, Finance, Delta	All salaries and wages in the functional expense category instruction (see instructional expenditure). Includes all compensation for services to all employees in the colleges, schools, departments, and other instructional divisions of the institution—full-time and part-time faculty, staff, regular employees, and student employees—and for departmental research and public service that are not separately budgeted. It includes compensation for academic instruction, occupational and vocational instruction, community education, preparatory and adult basic education, and remedial and tutorial instruction conducted by the teaching faculty for the institution's students.	Comparison with faculty variables
Completions for full-time first-time cohort	IPEDS, Grad-uation Rates, Delta	Number of first-time, full-time degree/certificate-seeking students in 2007 starting-year cohort of who started and graduated from the same institution within 150% of normal time (within six years for 4-year institutions, and within three years for 2-year institutions). See IPEDS Graduation Rate publication, nces.ed.gov/pubs2017/2017046.pdf.	Secondary student outcome variable, measure of educational output
Full-time first-time cohort	IPEDS, Grad-uation Rates, Delta	Number of students in 2007 starting-year cohort of full-time first-time degree/certificate-seeking students. This adjusted cohort excludes students who suffered from permanent disability, passed away, or began service in the armed forces (including calls to active duty), a foreign aid service of the federal government (such as the Peace Corps), or an official church mission. See IPEDS Graduation Rate publication, nces.ed.gov/pubs2017/2017046.pdf	Size variable for FTFT completions, used in calculating graduation rate percentages
Completions for FSA cohort	FAFSA, NSLDS, SC	Number of Federal Student Aid receiving students in 2007 FSA cohort who completed a degree within 6 years at any institution (by 2012-13, or award year 2013). Calculated as the FSA Cohort multiplied by the sum of three completion rate	Primary student outcome variable,

		percentages: graduates from original institution, a different 4-year institution, or a different 2-year institution. See Scorecard Documentation pg. 22 (link) and Presidential Report pg. 29 (link).	measure of educational output
FSA cohort (6yr)	FAFSA, NSLDS, SC	Number of students in 2007 starting-year cohort of Federal Student Aid-receiving students. This includes all Federal Student Loan and Pell grant recipients (except for students who only received Pell grants prior to 2012, when reporting became required). Students are placed in the cohort year of first enrollment, counting backwards from the first year they received federal aid by their number of years of academic progress, up to two years, according to self-reported grade level. NSLDS attributes students only at the school they first received federal aid, to avoid double counting transfer students. See Scorecard Documentation pg. 22 (link) and Presidential Report pg. 29 (link).	Size variable for FSA completions, used in calculating graduation rate percentages
Net Assets	IPEDS, Finance, Delta	"Total net assets is the sum of net assets invested in capital assets, net of related debt, restricted-expendable net assets, restricted-nonexpendable net assets, and unrestricted net assets. It can be calculated as the difference between total assets and total liabilities." – copied from Delta Dictionary	Used as an alternative size variable in Figure C-1
All Employees	IPEDS, Fall Staff, Delta	Total number of employees, including full-time and part-time faculty (see Total Faculty) as well as full-time and part-time staff. Staff include executive/administrative and managerial employees, technical and paraprofessional employees, clerical and secretarial employees, skilled crafts employees, service/maintenance employees, and other professionals.	Used as an alternative size variable in Figure C-1
Out-of-state tuition price	IPEDS IC, SC	The tuition and fees charged by institutions to full-time undergraduate students who do not meet the state's residency requirements.	Figure 4 of main text
Normalized SAT Scores	Brook-ings, IPEDS	These data come from IPEDS Institutional Characteristics originally, but the Brook-ings value added project normalizes them and imputes the values for schools which do not report them. These data are for the main campus of grouped institutions.	Figure 4 of main text
Earnings	MRC	The average 2014 earnings of students born in 1984 who attended the school between ages 19 and 22 (see Appendix A for more detail).	Figure 4 of main text
R&D revenue	HERD Survey, NSF	The sum of R&D expenditures in FY 2013 that came from either the U.S. federal government, state and local governments, businesses or non-profit organizations. Omits own institutional funds.	Comparison with IPEDS research expenditure, Appendix F

Appendix C: Total enrollment headcount as the underlying size variable

We used total enrollment headcount as the fundamental measure of scale for the entirety of our analysis. We chose this measure of size because our focus is on the university as a fundamental service to the student and this measure allows us to naturally compare inputs and outputs relative to the student along with the tradeoffs associated with other functions of the university.

Non-enrollment variables as the underlying size variable

Figure C-1

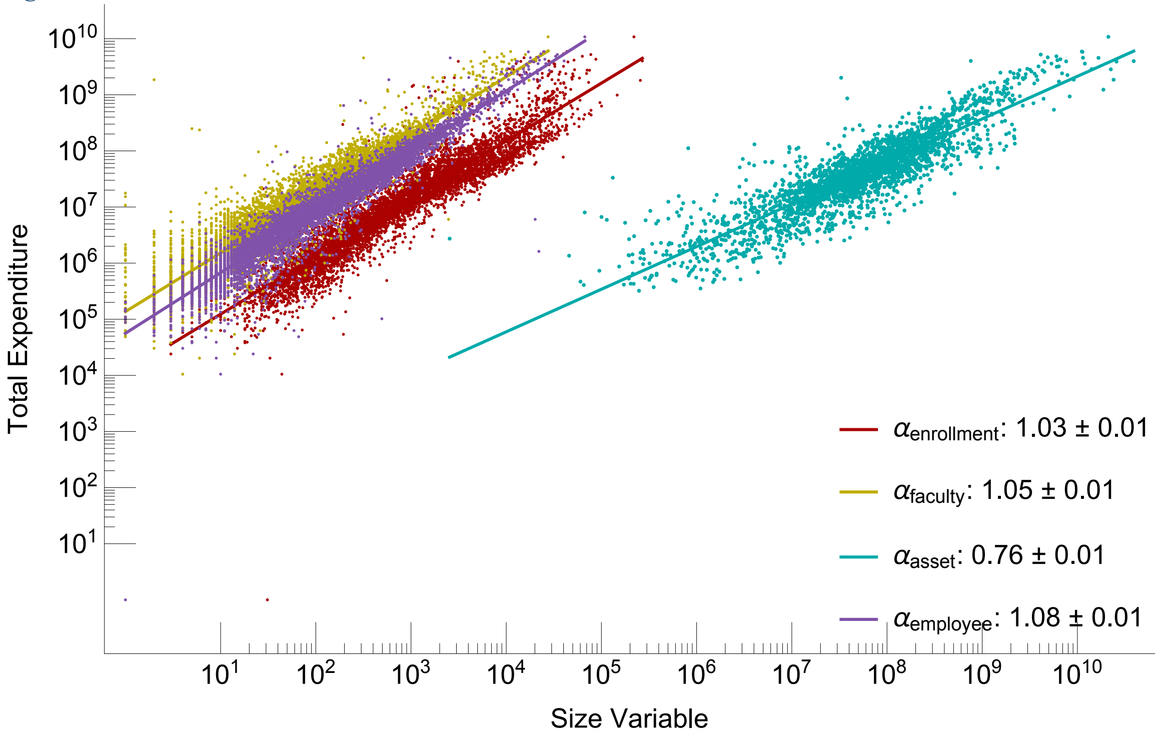

Figure C-1 shows the exponents, size range and variance of total expenditure regressing on different size variables, in which $\alpha_{enrollment}$, $\alpha_{faculty}$, α_{asset} and $\alpha_{employees}$ refers to total enrollment, total faculty, net assets and total employees respectively.

Other enrollment variables as the underlying size variable

Instead of total enrollment, we could have used full-time equivalent (FTE) enrollment, to account for the fact that many students are not full-time students. There are pros and cons to each measure. Total enrollment is a good measure of the fixed cost associated with administering and hosting a student, but an imperfect measure for the variable cost of the time spent on instruction. Total enrollment is also a good measure of the number of students interacting with the institution and with each other and thereby generating possible network economies. However, FTE is a

better measure for any input or output variable that is sensitive to the time spent by students on campus, such as teaching expenditure and tuition costs. For robustness, in Table C-1 we thus reproduce Table 2 of the main text using FTE as our measure of size (we don't include student outcomes here since they are analyzed as a function of cohort size instead of total enrollment). We see that coefficients are very similar to and share overlapping confidence intervals with those in Table 2, exceptions noted below.

Table C-1

variable name	Public Research Universities	Private Research Universities
Instruction	y = **1.22**±0.09x + 7.05±0.89; N:159	y = **1.45**±0.15x + 5.69±1.36; N:102
Tuition Rev	y = **1.19**±0.08x + 7.3±0.76; N:159	y = **1.17**±0.08x + 8.48±0.68; N:102
Research	y = **1.59**±0.29x + 2.4±2.89; N:159	y = **1.93**±0.59x + -0.3±5.37; N:84
Government Grants and Contracts	y = **1.33**±0.22x + 5.31±2.18; N:159	y = **2.02**±0.45x + -1.18±4.02; N:100
Maintenance	y = **1.08**±0.1x + 8.04±0.95; N:159	y = **1.34**±0.15x + 6.49±1.38; N:102
Total Faculty	y = **1.17**±0.08x + -3.66±0.79; N:159	y = **1.17**±0.12x + -3.21±1.09; N:102
Instructor Pay	y = **1.21**±0.09x + 6.61±0.86; N:159	y = **1.41**±0.14x + 5.61±1.28; N:102

variable name	State Colleges	Community Colleges
Instruction	y = **0.95**±0.04x + 9.22±0.31; N:379	y = **0.86**±0.02x + 9.71±0.14; N:903
Tuition Rev	y = **1.11**±0.05x + 7.81±0.42; N:376	y = **0.95**±0.03x + 8.55±0.25; N:901
Research	y = **0.98**±0.27x + 4.93±2.3; N:302	y = **0.12**±0.31x + 10.43±2.47; N:83
Government Grants and Contracts	y = **0.7**±0.1x + 9.94±0.89; N:377	y = **0.76**±0.05x + 9.13±0.38; N:895
Maintenance	y = **0.85**±0.05x + 9.79±0.4; N:380	y = **0.93**±0.02x + 8.79±0.19; N:903
Total Faculty	y = **0.91**±0.03x + -1.7±0.29; N:380	y = **0.88**±0.02x + -1.42±0.18; N:908
Instructor Pay	y = **0.95**±0.03x + 8.72±0.3; N:379	y = **0.87**±0.02x + 9.13±0.14; N:902

variable name	Non-profit Private Colleges	Professional Schools	For-profit Colleges
Instruction	y = **1.**±0.02x + 9.01±0.16; N:1356	y = **0.95**±0.02x + 8.65±0.13; N:2140	y = **0.94**±0.04x + 8.74±0.26; N:615
Tuition Rev	y = **1.15**±0.02x + 8.53±0.15; N:1344	y = **1.07**±0.03x + 8.98±0.13; N:2137	y = **0.98**±0.03x + 9.98±0.2; N:614
Research	y = **0.66**±0.13x + 8.21±0.98; N:338		
Government Grants and Contracts	y = **0.88**±0.08x + 7.03±0.54; N:993		
Maintenance	y = **0.89**±0.02x + 9.82±0.16; N:1354		y = **0.78**±0.14x + 10.42±0.84; N:17
Total Faculty	y = **0.88**±0.02x + -1.26±0.13; N:1362	y = **0.73**±0.02x + -1.15±0.1; N:2221	y = **0.84**±0.04x + -1.08±0.22; N:646
Instructor Pay	y = **0.98**±0.02x + 8.69±0.15; N:1355		y = **0.96**±0.23x + 8.38±1.41; N:17

Table C-1 shows 1) the fitline equations for scaling relationships of key variables versus FTE enrollment and 2) the number of schools qualified for the fitline calculation. Scaling exponents are in bold. These table is meant for comparison with Table 2 of the main text.

There are a few instances where the exponents differ when using total enrollment or FTE differ such that their confidence intervals do not overlap, or they do not exhibit the same overall scaling behavior.

1) Tuition scaling for state colleges changes from 1.04 ± 0.06 versus total enrollment to 1.11 ± 0.05 versus FTE, for community colleges changes from 0.89 ± 0.04 to 0.95 ± 0.04, putting some nuance on our conclusion that at scale, the affordability of these schools remains constant or increases. Indeed, the sublinear scaling shown in Table 2 may be a result of the cheaper tuition for part-time students and their increasing population in state/community college. We note that the sublinear scaling of public funding is extremely robust. Hence, the economies of scale of community and state colleges are in large part associated with (or a consequence of) this reduction in public funding. It is a little less clear whether they lead to a reduction in the costs to students.

2) Using FTE shifts two scaling exponents enough to change their overall scaling behavior, because of their proximity to the somewhat arbitrary boundaries that we use to define linear scaling, from 0.95 to 1.05. However, each of these changes is slight and occurs well within the original exponent's confidence intervals. The changes do not alter our comparisons of scaling relationships within or between sectors, in the main text.
   a) Teaching expenditures at state colleges changes from 0.9 ± 0.04 to 0.95 ± 0.04
   b) Faculty pay at state colleges changes from 0.91 ± 0.04 to 0.95 ± 0.03
   c) Maintenance expenditures for community colleges changes from 0.88 ± 0.02 to 0.93 ± 0.02.

Appendix D: University sector definitions and outlier analysis

Our main findings rely upon grouping universities into distinct sectors for comparison, so the exact definition of sectors crucially determines the results and their interpretation. In this Appendix, we will discuss (1) the detailed definitions of seven sectors we used in the main text, (2) rationale for merging for profit 2yr and 2yr- universities, and (3) different cases of outlier institutions, how they impact the scaling of their sector, and why we exclude or include them.

Seven sector definitions

Our sector classification in Table D-1 encompasses the main institutional archetypes typically discussed in US higher education (Bok 2013). As mentioned in the main text, the seven sectors we identify are drawn conventionally along the dimensions of control, level, and research activity. Control categories are public, private non-profit, and private for-profit, each representing a different "business model." Level categories are 4-year and higher (4yr+), 2-but-less-than 4-year (2yr), and less than 2-year (2yr-), based on the typical time to completion of the highest-offered degree or certification at the institution. Of the resulting nine sectors, we excluded the three with fewer than 100,000 students each, comprising together barely half a percent of total enrollment in the delta dataset. We combined private for-profit 2yr and 2yr- sectors because they share a professional focus, and exhibit almost exactly the same scaling behavior, as shown below in Table D-2. As for research, we observe that its presence as a primary institutional objective, in addition to education, entails fundamentally different financial flows, distinct scaling in all key variables, and therefore different institutional type. At the greater than four-year level we separated research and non-research universities using the 2010 Carnegie Classification for both public and private non-profit institutions. We grouped all three tiers of research activity into research sectors, and the remaining four-year masters and baccalaureate Carnegie Sectors into non-research. We excluded all Puerto Rican and U.S. Territory schools, and medical schools classified as non-research four-year institutions.

Table D-1

Sector name	Control	Level	**Examples** (from one metro area for comparability)
Public Research Universities	Public	4yr+, Doc	UCLA
Private Research Universities	Private N-P	4yr+, Doc.	USC University of Southern California
State Colleges	Public	4yr+	CAL STATE LA California State University, Los Angeles
Community Colleges	Public	2yr	LACC Los Angeles City College
Non-Profit Private Colleges	Private N-P	4yr+	Pomona College; Fuller Theological Seminary
For-Profit Colleges	For-Profit	4yr+	Platt College — it's all about your future —
Professional Schools	For-Profit	2yr, 2yr-	american career college; CHAC California Healing Arts College

It is useful to understand how the size ranges of sectors compare, prior to conducting scaling analysis. The box-and-whisker plot below, Figure D-1, shows the distribution of enrollment in different sectors. Note how the middle two quartiles of the sectors' size ranges hardly overlap, though their full ranges consistently do.

Figure D-1

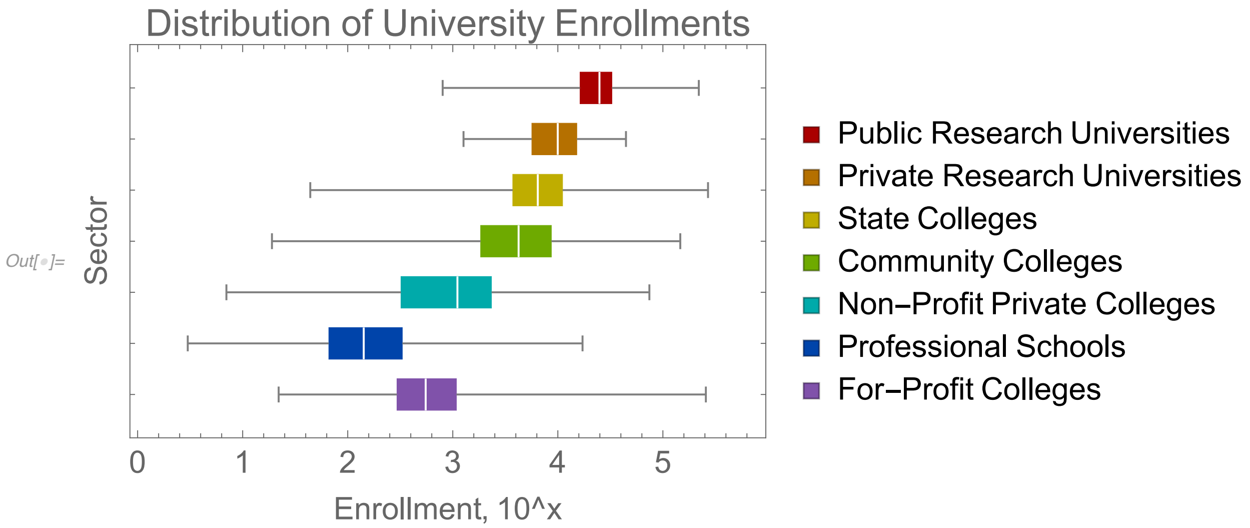

Merging for-profit 2yr and 2yr- universities to create professional school sector

We noticed several important similarities between private for-profit 2yr universities and private for-profit 2yr less universities. The two sectors share a vocational focus, comprised mostly of programs in cosmetology, nursing, and technical areas. Likely by consequence, they exhibit very similar scaling behavior. The confidence intervals of their respective scaling exponents for key

variables always overlap, as shown in the first two columns of Table D-2. Furthermore, the scaling intercepts are only ever slightly different between the two sectors and exist in similar size ranges (though the less-than-2yr schools are generally smaller). For these reasons, it makes sense to consider whether they constitute a single institutional sector. We tested this directly by regressing on the entire sample of 2142 schools and comparing the scaling results of the individual sectors to these new results, in column three of Table D-2.

Table D-2

variable name	Private for-profit 2yr	Private for-profit 2yr-	Professional Schools
Total Revenue	y = **1.03**±0.04x + 9.32±0.21; N:799	y = **0.98**±0.03x + 9.39±0.16; N:1343	y = **1.03**±0.02x + 9.22±0.12; N:2142
Total Expenditure	y = **1.05**±0.04x + 9.2±0.23; N:799	y = **1.01**±0.03x + 9.16±0.16; N:1343	y = **1.06**±0.02x + 9.±0.13; N:2142
FSA Completions	y = **0.93**±0.07x + -0.07±0.49; N:121	y = **1.05**±0.09x + -0.86±0.6; N:73	y = **0.96**±0.06x + -0.26±0.42; N:168
First-time full-time Instructor Pay	y = **1.02**±0.02x + -0.64±0.1; N:731		y = **1.02**±0.02x + -0.64±0.1; N:731
Total Faculty	y = **0.78**±0.03x + -1.32±0.17; N:824	y = **0.67**±0.03x + -1.06±0.13; N:1398	y = **0.76**±0.02x + -1.35±0.1; N:2222
Student Tuition	y = **1.09**±0.05x + 8.47±0.26; N:759	y = **1.06**±0.05x + 8.36±0.26; N:1217	y = **1.11**±0.03x + 8.23±0.18; N:1976

Table D-2 shows the exponents of key variables in three sectors: private for-profit 2yr and private for-profit 2yr less, and their combined sector (professional schools). Each table entry is a fitline equation in log-log space and the exponent value is bolded. FSA completions data are not used; see Appendix G for our explanation.

We see that total expenditures in the combined sector scale with an exponent exceeding 1.05, which qualifies it as superlinear, despite both constituent sectors exhibiting linear scaling ($0.95 \leq b \leq 1.05$) for this variable. However, all confidence intervals overlap, and in the main text we consider total expenditures of professional schools to scale linearly or very slightly superlinearly, with respect for the arbitrariness of the 1.05 boundary. Thus we find the combined sector overall displays the same scaling as the 2yr and 2yr- sectors, so we use it throughout our research under the name professional schools.

Introduction to outlier analysis

In our approach, as we group universities into sectors and run regressions on them, scaling exponents are not only our results of interest, but help us differentiate between university sectors. However, our groupings are legitimate insofar as the residuals of the data are uniformly distributed across size. In other words, curvature or outliers that are heavily concentrated at the head or the tail of the scaling fitline can disproportionately influence the exponent value, compared to those located in the middle of the domain. Besides challenging the robustness of our analysis, outliers themselves also reveal the underlying constraints of the system and potential opportunities to break them. Therefore, we find it crucial to identify outliers and investigate what they do differently to leverage synergies or efficiencies at scale, and thereby strengthen our understanding of scaling mechanisms and university strategies.

By filtering out data that have high residuals, we identified four main groups of outliers, each explained as the special case sections below. We carefully scrutinized these groups in two ways. On one hand, we compared the scaling of these subsets of data to all other universities in the sector, using total expenditure versus total enrollment as our test case. On the other, we confirmed that we have theoretically sound reasons for excluding or including them.

We summarize our treatment of outlier institutions here:

- Universities with medical schools or hospitals are excluded from non-research 4yr+ sectors (state colleges and non-profit private colleges)
- Universities with medical schools and hospitals are included, in both public and private non-profit domains
- Rockefeller University is excluded from the private research university sector
- Online-only schools and schools based in U.S. Territories are excluded from all sectors

We recognized that there exist other institutional types that tend to have high residuals, such as some stand-alone law schools, art and theater institutes, business schools and grouped schools that combine multi-sector colleges into one. However, we decided to not analyze them in-depth with the belief that their population is too small to significantly skew our outcomes.

Outlier analysis helped with finer tuning to isolate sectors of comparable institutions, explained fully below. Though revealed through the analysis, we feel well justified in removing these groups on purely theoretical grounds. Nonetheless, a more holistic approach might account for a spectrum of institutional types: for example, private research universities range from having no medical school or maintaining a distant affiliation to having one completely integrated into the greater university. To determine natural classes of institutions, future analyses may wish to perform *a priori* algorithmic classification schemes.

Special case: research universities

After other exclusions in this appendix, a total of 268 Delta schools are in the three tiers of "doctoral universities" set forth by the 2010 Carnegie Classification (Carnegie Foundation for the Advancement of Teaching, 2011). These three tiers of research activity are Tier 1 – Research Universities (very high research activity), Tier 2 – Research Universities (high research activity), and Tier 3 – Doctoral/Research Universities. This combined class of universities includes only 4yr+ schools that awarded twenty or more research/scholarship doctorates between Fall 2008 and Spring 2009, according to the IPEDS Completions Survey. We term these schools "Research Universities." By control, 160 are public, 102 are private non-profit, and 6 are private for-profit.

Shown in Figure D-2 and Figure D-3 respectively, public and private research universities (circled in green) exhibit markedly different scaling from their non-research counterparts. The clusters of research universities cover most of the upper-end curvature above the fitline, and exhibit superlinear scaling very different from that of non-research universities. The same patterns are not observed for the six for-profit research universities, which fall neatly around the scaling relationships for all for-profit 4yr+ schools, and do not change the scaling when removed.

The research cluster also overlaps with medical schools at larger size, especially those typically associated with prestige and extensive facilities. It is clear that both research and medical schools can explain the outlier curvature at the tip of the graph, and separating them can improve the robustness of scaling model on the rest of the data—but why do we choose research as the breaking point for sector definition rather than medical schools? We argue that medical schools within their own set can have very different internal structures depending on whether they are stand-alone institutions or are hosted within big research universities. Meanwhile, we reason that

research universities with and without medical schools are more likely to share institutional structure, whatever that may consist of. Furthermore, research is a widely accepted sector divider when discussing American universities, informally and in the higher education literature (Bok 2013, Crow and Dabars 2015). This allows us to approach prevailing hypotheses about scale in higher education. Therefore, we are confident in dividing the public 4yr+ and private non-profit 4yr+ universities each into research and non-research sectors. We decided to include research universities that have a medical school or hospital, because their medical facilities cannot clearly be separated from their institution-wide research activities.

Figure D-2

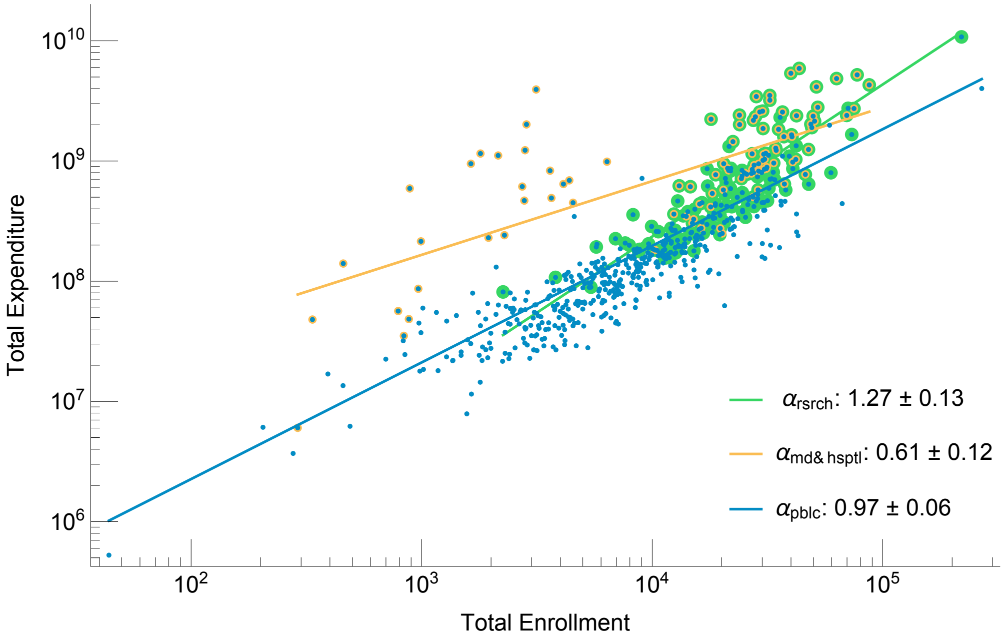

Figure D-2 shows the overlap between research and medical schools among public 4yr+ universities. Scaling exponents in the order α_{rsrch}, $\alpha_{md\&hsptl}$, α_{pblc}, refer to schools that are classified as research universities, schools that grant medical degrees or have hospitals, and all universities in the public 4yr+ sector.

Figure D-3

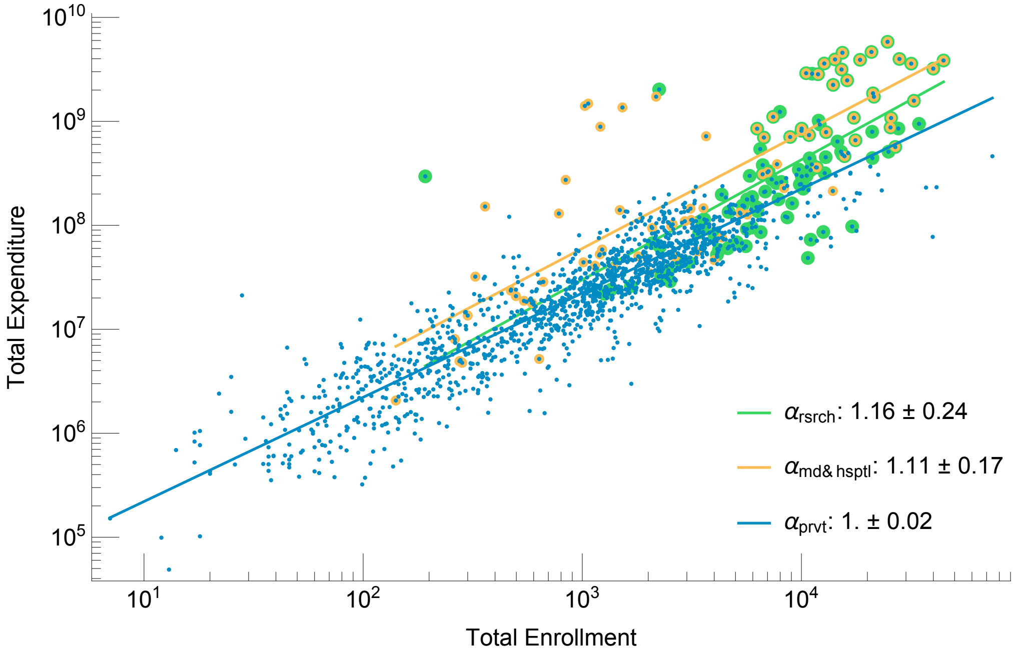

Figure D-3 shows the overlap between research and medical schools among private 4yr+ universities. Scaling exponents in the order α_{rsrch}, $\alpha_{md\&hsptl}$, α_{pblc}, refer to schools that are classified as research universities, schools that grant medical degrees or have hospitals, and all universities in the private 4yr+ sector.

Special case: universities that grant medical degrees or have hospitals

There are 162 schools that grant medical degrees and 84 schools that have hospitals in our Delta dataset. Most of these universities are in public and private non-profit 4yr+ realm, and may or may not have a research component. Moving forward, we simply refer to them as medical schools and hospitals.

From Figure D-4 and Figure D-5 we can see that medical schools (in red or yellow color) as a subset of data have a much higher normalization constant, which means that their total expenditure and revenue is also higher on average. In particular, universities that have hospitals or both tend to have even larger residuals than those who only have medical degrees, implying that it is the nature of medical field practice to expand financial flow. Furthermore, many medical schools/hospitals enroll only graduate students, spend disproportionately large amounts per student, and often function primarily as free-standing medical institutions rather than as institutes of postsecondary education. For all the reasons given above, we are confident in excluding these institutions from non-research 4yr+ private colleges and state colleges. Removing medical schools does not impact the scaling exponent of total expenditure for non-profit private colleges, but increases state colleges’ scaling exponent from 0.69 to 0.84 with a narrower confidence interval for the exponent.

We only apply these exclusions to sectors of non-research colleges. As mentioned, we still include research universities that report having a medical school or hospital.

Figure D-4

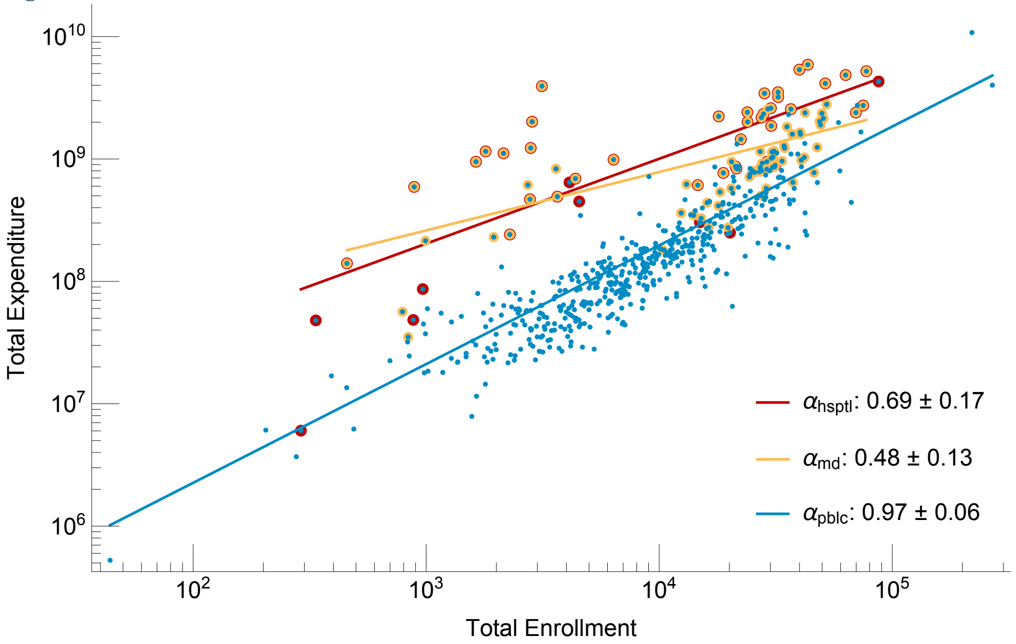

Figure D-4 shows the overlapping attributes of medical degrees and hospitals in Public 4yr+ universities. Scaling exponent in the order of α_{hsptl}, α_{md}, α_{pblc}, refers to schools that have at least one hospital in Public 4yr+ sector, schools that grant medical degrees in Public 4yr+ sector, and all universities in Public 4yr+ sector.

Figure D-5

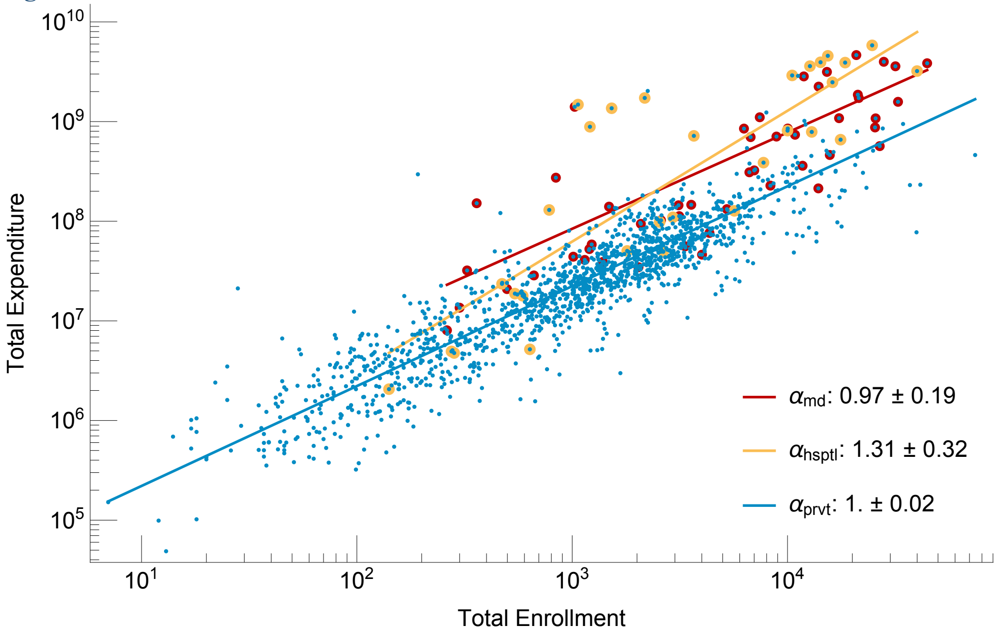

Figure D-5 shows the overlapping attributes of medical degrees and hospitals in Private 4yr+ universities. Scaling exponent in the order of α_{hsptl}, α_{md}, α_{prvt}, refers to schools that have at least one hospital in Private 4yr+ sector, schools that grant medical degrees in Private 4yr+ sector, and all universities in Private 4yr+ sector.

Special case: Rockefeller University

In the private research university sector, Rockefeller University stands out as the biggest outlier with a normalized residual of 5.22 standard deviations above the fitline when it is not included. Less than 200 graduate students comprise its total enrollment, yet its expenditures total on the order of hundreds of millions. The exclusion of Rockefeller University from the dataset can significantly alters the scaling exponents of all variables of interest in the sector (see Figure D-6 and Table D-3), notably driving up the exponents of variables relevant to research and maintenance.

Figure D-6

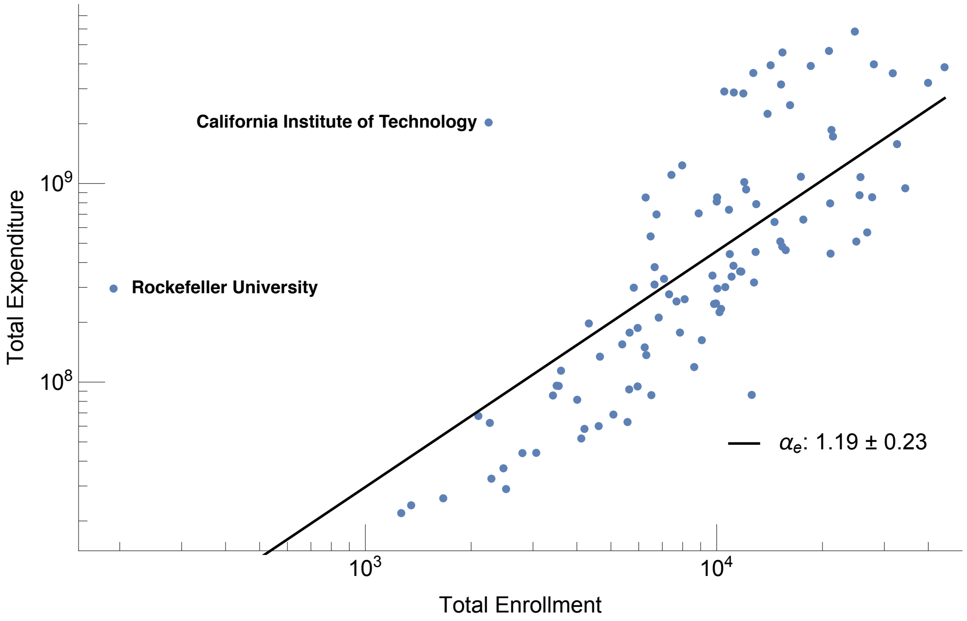

Figure D-6 shows the position of the two top outliers in the private research university sector, and the fitline calculated including them.

Table D-3

variable name	Private Research Universities without Rockefeller	Private Research Universities
Total Expenditure	y = **1.46**±0.22x + 6.5±2.04; N:102	y = **1.19**±0.23x + 9.±2.07; N:103
Total Revenue	y = **1.52**±0.24x + 6.15±2.15; N:102	y = **1.22**±0.24x + 8.88±2.2; N:103
Total Faculty	y = **1.18**±0.14x + -3.47±1.27; N:102	y = **1.**±0.14x + -1.8±1.31; N:103
Student Tuition	y = **1.22**±0.1x + 7.78±0.92; N:102	y = **1.22**±0.1x + 7.78±0.92; N:102
Government Student Grant Aid	y = **0.88**±0.21x + 7.98±1.91; N:99	y = **0.88**±0.21x + 7.98±1.91; N:99
Government Appropriation	y = **1.69**±1.24x + -1.11±11.75; N:21	y = **1.69**±1.24x + -1.11±11.75; N:21
Government Grants and Contracts	y = **1.94**±0.49x + -0.71±4.51; N:100	y = **1.46**±0.47x + 3.76±4.3; N:101
Donation Investment Rev	y = **1.69**±0.36x + 2.9±3.32; N:102	y = **1.31**±0.36x + 6.48±3.28; N:103
Auxiliary Rev	y = **1.98**±0.35x + 0.07±3.2; N:102	y = **1.58**±0.35x + 3.84±3.22; N:103
Instruction	y = **1.44**±0.18x + 5.53±1.64; N:102	y = **1.44**±0.18x + 5.53±1.64; N:102
Research	y = **1.75**±0.61x + 1.1±5.66; N:84	y = **1.15**±0.56x + 6.76±5.14; N:85
Public Service	y = **1.36**±0.46x + 2.95±4.25; N:61	y = **1.36**±0.46x + 2.95±4.25; N:61
Maintenance	y = **1.33**±0.18x + 6.41±1.65; N:102	y = **1.08**±0.19x + 8.71±1.73; N:103
Student Service	y = **1.09**±0.14x + 7.16±1.28; N:102	y = **0.92**±0.14x + 8.73±1.29; N:103
Grants and Auxiliary	y = **1.83**±0.37x + 1.05±3.35; N:102	y = **1.5**±0.35x + 4.12±3.22; N:103

Table D-3 shows the scaling exponents of various variables in the private research university sector, with and without Rockefeller University. Each entry is a fitline equation in log-log space and the exponent value is bolded.

The unique and special history of Rockefeller University justifies its special status and the exclusion from our scaling analysis. Established as The Rockefeller Institute for Medical Research in 1901 it was modeled after similar medical research institutions in Europe, especially the Pasteur Institute in Paris and the Robert Koch Institute in Berlin. Its goal was to advance biomedical research in the US by focusing primarily on basic science, but also on clinical applications. The latter began in earnest in 1910, with the establishment of Rockefeller Hospital. For the first decades of its existence the institute was research only. It recruited top researchers and gave them resources and freedom, thus following a similar path as the Kaiser Wilhelm Society (later Max Planck Society) with its famed Harnack Principle, named after the first President of the KWG, Adolf von Harnack. It is also important to note that Abraham Flexner, the younger brother of Rockefeller's first president, Simon Flexner, is well known for his detailed comparative evaluation of higher education and especially medical research and teaching, which undoubtedly influenced the unique trajectory of The Rockefeller Institute. As part of their recruitment strategy, Rockefeller attracted top researchers from Europe and the US, including many refugees.

Rockefeller awarded its first Ph.D. degree only in 1954 and currently operates a very small graduate program. It has a faculty of 82 largely independent researchers who supervise a staff of more than 1500 researchers, postdocs, clinicians and technicians and only 175 graduate students (Ph.D. and M.D./Ph.D.). Perhaps unsurprisingly, the institute's mission statement, unchanged "since 1913," makes no mention of education (https://www.rockefellerfoundation.org/about-us/).

As such, based on its unique history and organizational structure we can safely exclude Rockefeller from a comparative analysis, as it is truly *sui generis*. In addition, we checked whether the undergraduate share of enrollment at universities is correlated with the variance (residuals) because Rockefeller has 100% grad students. One could argue that Rockefeller is less

like a research university and closer to a stand-alone medical school which would also be composed only of graduate students and focused on biomedical research and practice. Figure D-7 and Figure D-8 confirm that Rockefeller and some stand-alone medical schools all have similarly high expenditure residuals and negligible undergrad enrollment. For all public 4yr+ universities, outlier schools also have high graduate student enrollment, but for private 4yr+ universities, the correlation between percentage of undergraduate enrollment and residuals is weak and noisy—many theological seminaries and other special-focus institutions are also graduate-only, but unlike medical schools, perfectly conform to the rest of the sector's scaling. Therefore, we conclude that undergraduate share of enrollment, or presence of undergraduates, is insufficient evidence to exclude Rockefeller and/or other graduate-only institutions.

California Institute of Technology (CalTech) is the second-most significant outlier in the private research university sector. We know that its enormous amounts of total revenue and expenditure stem from the Jet Propulsion Laboratory (JPL), a federally funded research center that it houses. Much like medical schools, such university research and development centers exist on a spectrum, from practically independent operations to deeply embedded fixtures in the greater university. We have no comprehensive, specific method to determine which labs are separable from their institution-wide research activities. Consequently, we keep CalTech and all such "parent" institutions in the dataset (see Appendix E).

Figure D-7

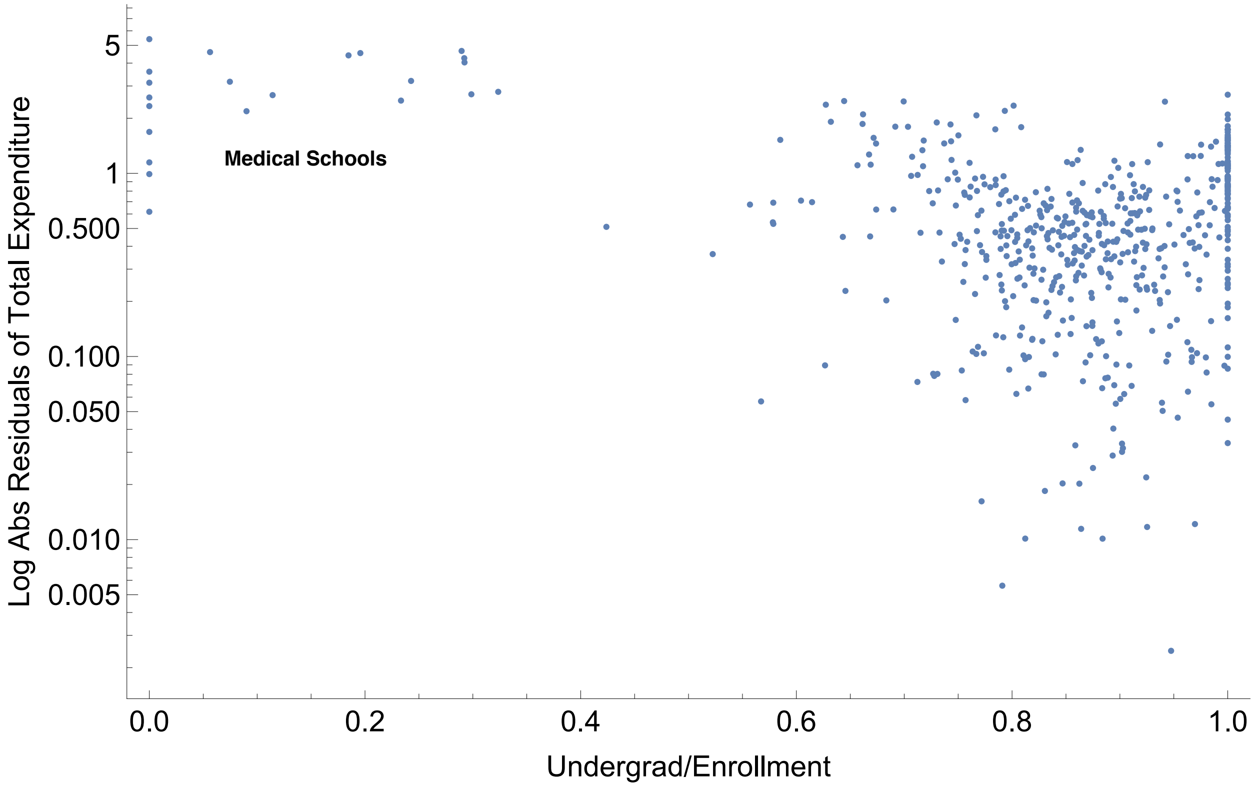

Figure D-7 shows log-normalized absolute residuals of total expenditure scaling, versus (linear) percentage of undergrad enrollment for public 4yr+ universities including both research and non-research sectors. The fitline for residuals is calculated excluding graduate student-only schools.

Figure D-8

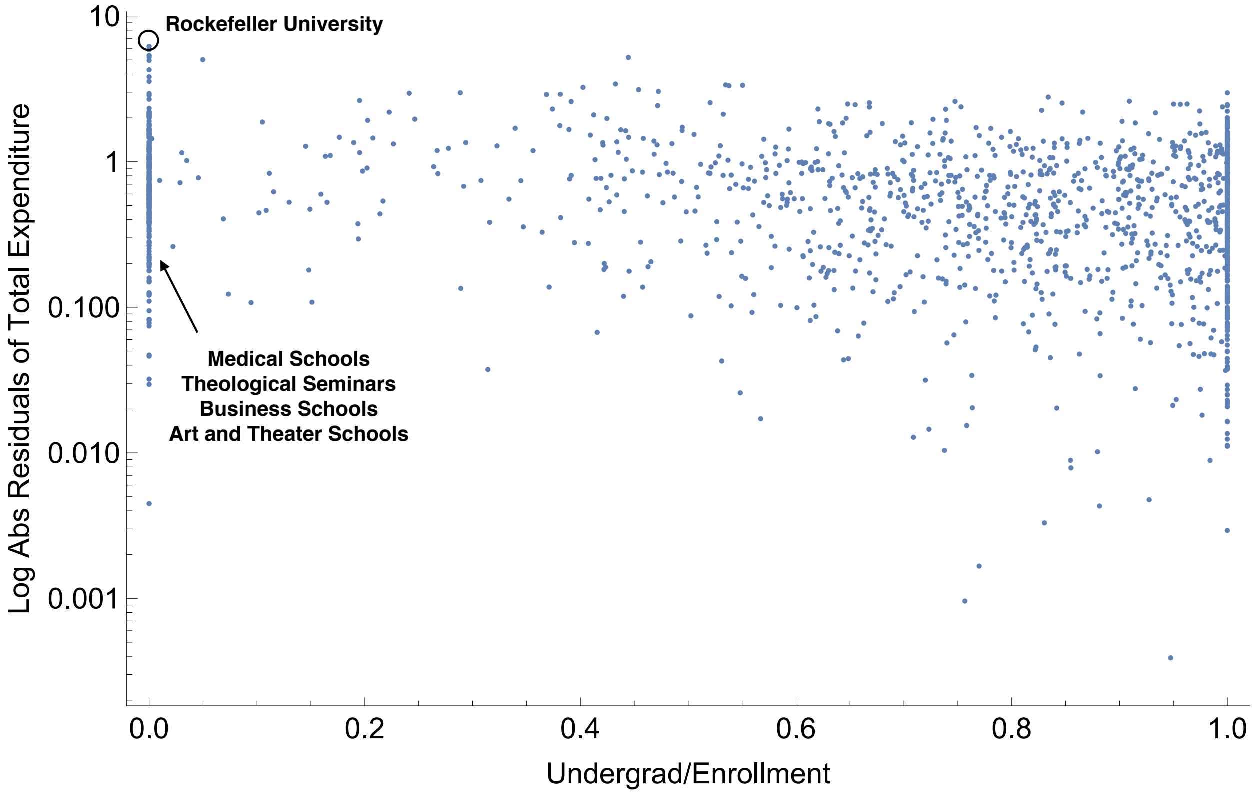

Figure D-8 shows log-normalized absolute residuals of total expenditure scaling, versus (linear) percentage of undergrad enrollment for private 4yr+ universities including both research and non-research sectors. The fitline for residuals is calculated excluding graduate student-only schools.

Special case: online schools

Online programs are popular targets for universities in recent years accompanied by the rise of online education technology. Intuitively, online programs can exhibit large economies of scale because once the initial cost of establishment is stabilized, universities can potentially attract more out-of-state and non-US students and invest less physical facilities per student without affecting the quality of education with scale. We used the variable "DISTANCEONLY" in College Scorecard to distinguish schools that are entirely online. Out of the 32 online schools, 22 are for-profit 4yr+ colleges, while another seven are non-profit private colleges.

For both sectors, online schools scale sublinearly and in particular, scale even more sublinear than all the data in Non-profit private college sector. Because we set a threshold of fifteen points to run a regression, we only show the graph for the for-profit 4yr+ sector. Including online programs does not change the scaling exponents because these schools compose a small percentage of all schools considered in the regression. However, we still decided to set aside online schools as they display a distinct scaling relationship and function very differently than universities with in-person campuses, and because their coverage in our dataset is too sparse to

draw conclusions; focused research on the scaling behavior of online schools or programs would be a valuable extension of the work presented here.

Figure D-9

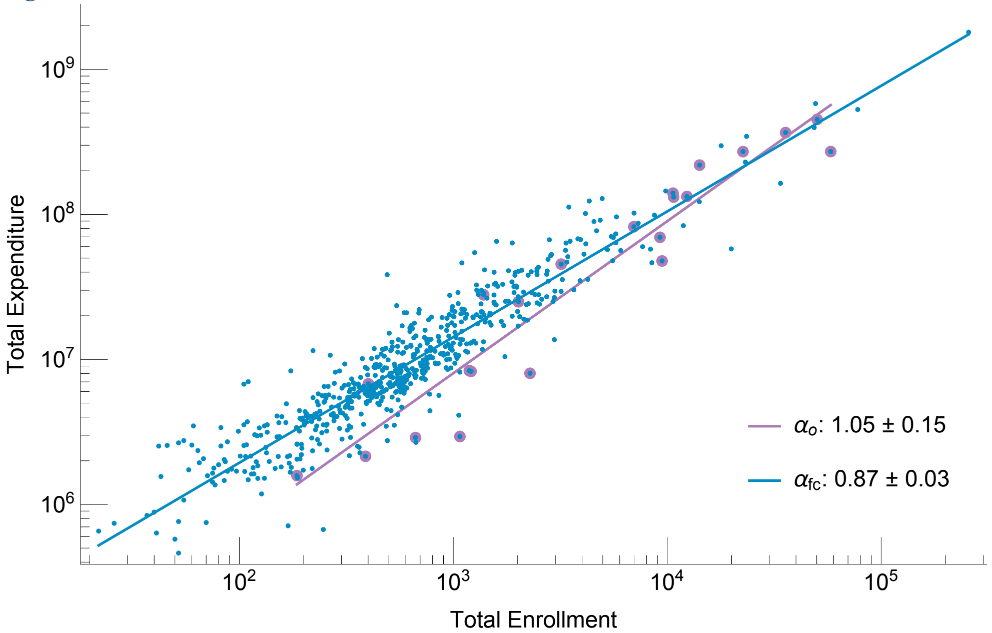

Figure D-9 shows the entirely online schools for for-profit 4yr+ universities. Scaling exponents, in the order of α_o and α_{fc}, refer to online schools in the for-profit college sector and to all schools in the sector.

Special case: U.S. territories universities

Similar to online programs, we also excluded universities located in U.S. territories from all sectors, including those at Puerto Rico and the Pacific Islands. We assume their economies and higher education system are qualitatively different, with much lower costs of living and labor than the in the fifty states. 94 out of 132 these schools are private non-profit colleges or professional schools, which exhibits sublinear scaling in their own sectors (Figure D-10 and Figure D-11).

Figure D-10

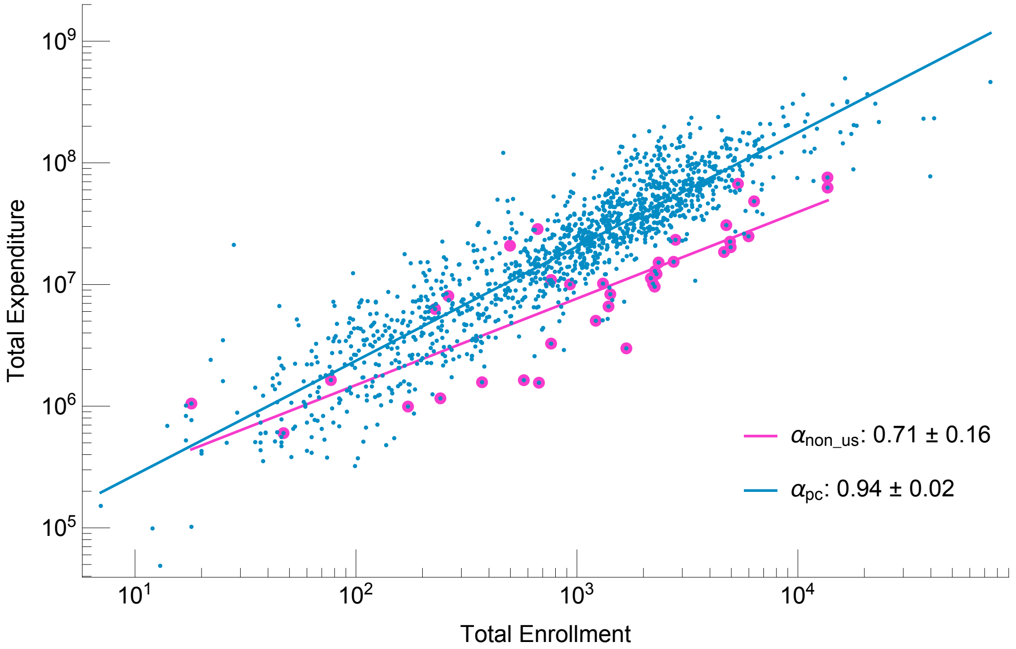

Figure D-10 shows the U.S. territories universities in the private non-profit college sector. Scaling exponent in the order of α_{non_us} and α_{pc} refers to U.S. territories colleges in the private non-profit college sector and all schools in the private non-profit college sector.

Figure D-11

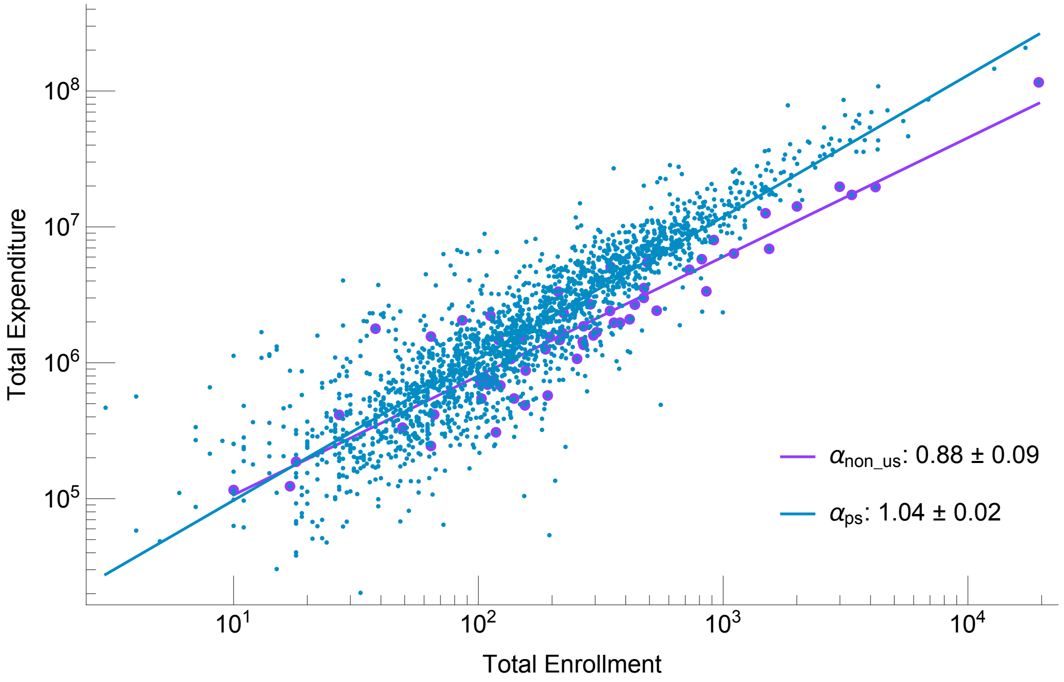

Figure D-11 shows the U.S. territories universities in the professional schools sector. Scaling exponent in the order of α_{non_us} and α_{ps} refers to U.S. territories, colleges in the professional school sector, and all schools in the professional schools sector.

Special case: liberal arts colleges

It should be noted that in our analysis the non-profit private colleges sector contains a fairly large amount of institutional diversity. This sector is defined as all private non-profit colleges in non-research classes of four-year universities, per the 2010 Carnegie Classification. These classes include Master's Colleges and Universities, Baccalaureate Colleges, Baccalaureate/Associate's Colleges, the entire swath of Special Focus Institutions (noting that we exclude medical schools), and the few universities outside the classification system. A notable example includes schools that award doctoral degrees such as the Rocky Mountain University of Health Professions. However, an important subcategory within this sector, and one which represents a well-known institutional archetype of US education, are the "liberal arts" colleges. It is possible to restrict our analysis within this broader sector to only Baccalaureate Colleges within the Carnegie Classification. Doing this we find different scaling relationships than the overall sector (Table D-4) where, most notably, the baccalaureate colleges have superlinear scaling for expenditure on instruction and faculty pay.

Table D-4

Out[ ]//TableForm=

sector name	Non-profit Private Colleges	Baccalaureate Colleges
Instruction	0.99 ± 0.02	1.14 ± 0.07
Student Tuition	1.17 ± 0.03	1.23 ± 0.08
Research	0.65 ± 0.14	0.93 ± 0.5
Government Grants and Contracts	0.85 ± 0.08	0.58 ± 0.2
Maintenance	0.89 ± 0.02	0.93 ± 0.07
Total Faculty	0.89 ± 0.02	0.96 ± 0.05
Instructor Pay	0.98 ± 0.02	1.1 ± 0.06
FSA Completions	0.99 ± 0.04	0.94 ± 0.08
First-time full-time	1.09 ± 0.02	1.15 ± 0.04
Mid-career earnings	1.18 ±0.04	1.15 ± 0.06

Appendix E: Delta Cost Project grouping of campuses and institutions

The Delta Cost Project groups schools in such a way that complicates both sector definitions and the institutional unit of analysis (Hurlburt, Peek and Sun 2017, Jaquette and Parra 2016). In their paper "The Problem with the Delta Cost Project," Jaquette and Parra advise against using Delta data for cross-sector comparisons. Here, we explain how Delta data groups universities and how it raises three main considerations for our scaling analysis: (1) possible statistical issues with all our scaling relationships due to the mismeasurement of university size; (2) matching data with the College Scorecard dataset for completions; (3) grouping universities across sectors. In total, we demonstrate that it has no major effect on our results.

Delta grouping of universities explained: multiple-campus institutions and multiple-institution systems

It is very common for universities to exist in multiple locations, or to maintain organizational ties with other universities. Consequently, "NCES allows certain institutions ('parent institutions') to report data for branch campuses or other affiliated institutions ('child institutions') for various IPEDS surveys," as stated on page 2 of the Delta data file documentation (Hurlburt, Peek and Sun 2017). This complicates IPEDS data, for two reasons: (1) institutions may choose to report

certain finance data at the parent level, while reporting other data separately by child institution, and (2) institutional parent-child relationships change when, for example, campuses are added, or schools merge. The Delta Cost Project maintains consistency of the data by grouping together universities that have reported any IPEDS data together at any time in the program's history, from 1987 to 2015.

The result is that Delta data collapses the data of many types of related institutions into a single entry with all the institutional characteristics (IPEDS UNITID, name, sector, SAT/ACT scores) of a particular school's location. We call such entries "Delta grouped" universities. They are denoted in the dataset by boolean variable 5 "isgrouped?" where 1 means grouped and 0 means not grouped or "ungrouped." See Delta Cost Project's Parent Child Master List for a crosswalk between 568 of the 616 Delta grouped institutions, and their respective 1,431 child institutions: https://deltacostproject.org/sites/default/files/ database/fy2015_parent-child-master-list.xls.

Table E-1 shows the extent of Delta grouping, measured by the percentage of schools that Delta groups and their share of total enrollment in each sector. Note that throughout the entire system, 10% of schools are grouped, but enroll 27% of students because they are all disproportionately large, and because many are large public research universities.

Table E-1

Sector name	N (schools)	Delta grouped N	Delta grouped %	Sector enrollment	Grouped enrollment	Grouped enrollment %
Public Research Universities	160	44	27.5	4 498 249	1 792 898	39.86
Private Research Universities	102	20	19.61	1 188 915	294 889	24.8
State Colleges	382	22	5.76	3 655 440	568 429	15.55
Community Colleges	908	101	11.12	6 517 164	1 830 210	28.08
Non–Profit Private Colleges	1373	81	5.9	2 524 604	390 504	15.47
Professional Schools	2230	214	9.6	695 753	211 222	30.36
For–Profit Colleges	647	86	13.29	1 230 372	318 829	25.91
All Universities	5802	568	9.79	20 310 497	5 406 981	26.62

Of these 1,431 child institutions, we are able to examine the 688 of them that appear in Scorecard, accounting for 1,696,276 undergraduate students (Scorecard undergraduate enrollment was used because neither total enrollment nor graduate enrollment are included in College Scorecard). The remaining 820 Scorecard schools that neither matched a Delta data entry nor a child institution wrapped into one account only for 143,179 undergraduate students.

Delta groups these 688 schools in two different ways, and a list identifying both cases accompanies the other data files used in our analysis. In the first case, there are 441 branch campuses, accounting for 405,075 undergraduate students, that Delta collapses under their respective main campuses (which holds the Program Participation Agreement or PPA with the U.S. Department of Education and shares its resulting designation as a Title-IV institution with the branch campuses). For example, Delta data combines the data from the four branch campuses of Arizona State University, located around the Phoenix metropolitan area, into a single observation: Arizona State University-Tempe Campus. Another example is Pennsylvania State University. Under the observation for the main campus, a top-tier research university, Delta

collapses data from all 22 campuses. They are spread throughout the state and range from localized associates- and bachelors-granting campuses to residential four-year campuses to a graduate professional school. Another example is the grouping of medical schools and hospitals with their associated research universities (see Appendix D).

In the second case of university grouping, Delta groups together some 247 Title-IV institutions accounting for 1,291,201 undergraduate students—into systems that are listed under one "linchpin" institution selected somewhat arbitrarily (Delta Cost Project 2011, Jaquette and Parra 2016). The most startling example is the University of Texas-Austin. It appears as the single largest public research university in Delta data, with total enrollment of about 219,000 students, because it combines values from all thirteen other Title-IV institutions in the University of Texas (UT) system such as UT Arlington, UT Brownsville and UT El Paso. (We note that not all university systems are grouped; for instance, each university in the University of California system appears separately in Delta data). Jaquette and Parra (2016) emphasize that such grouping of multiple-institution systems leads Delta to lack entries for many Title-IV institutions, particularly for public universities (missing 8%) as opposed to private non-profits (only 0.5%).

In the first case, of multiple-campus institutions, we do not have a clean-cut way of distinguishing how functionally separate the campuses are, and in the second case, we can be sure the grouped institutions can be considered functionally separate schools. Thus, to be conservative we must assume that any instance of a grouping in delta data erroneously combines functionally separate institutions.

Statistical problem with grouping, and its overall bearing on our scaling results

The grouping of arguably distinct data points artificially reduces the combined weight of the affected schools on the regression and creates an aggregate point with a new residual, located systematically up and to the right of where its constituent points would have been. This could bias the regression fit, the fitline slope, and the scaling result, all depending on the distribution of points within each grouping—along both vertical and horizontal dimensions.

To test the severity of this problem, we compare the scaling of all main variables with and without these delta-grouped schools. We check total revenues, total expenditures, tuitions, faculty, and faculty pay each versus enrollment, in every sector. We find that the removal of grouped schools has almost no effect on the scaling relationships. Not one of the exponents changes enough for its new confidence interval not to overlap with the old one.

Table E-2

'eForm=

variable name	Public Research Universities	Public Research UnGrouped	Private Research Universities	Private Research UnGrouped
Student Tuition	1.25 ± 0.13	1.34 ± 0.16	1.22 ± 0.1	1.24 ± 0.12
Government Student Grant Aid	0.94 ± 0.11	0.89 ± 0.14	0.88 ± 0.21	0.92 ± 0.26
Government Appropriation	1.07 ± 0.18	1.08 ± 0.24	1.69 ± 1.24	1.7 ± 1.55
Government Grants and Contracts	1.29 ± 0.23	1.27 ± 0.3	1.94 ± 0.49	1.9 ± 0.58
Donation Investment	1.81 ± 0.31	1.85 ± 0.42	1.69 ± 0.36	1.72 ± 0.4
Auxiliary	1.59 ± 0.23	1.63 ± 0.3	1.98 ± 0.35	1.99 ± 0.4
Total Revenue	1.29 ± 0.14	1.31 ± 0.19	1.52 ± 0.24	1.53 ± 0.27
Instruction	1.2 ± 0.1	1.23 ± 0.13	1.44 ± 0.18	1.43 ± 0.2
Research	1.52 ± 0.31	1.55 ± 0.38	1.75 ± 0.61	1.81 ± 0.66
Public Service	1.74 ± 0.32	1.88 ± 0.42	1.36 ± 0.46	1.24 ± 0.48
Maintenance	1.07 ± 0.11	1.04 ± 0.14	1.33 ± 0.18	1.36 ± 0.21
Student Service	0.95 ± 0.12	0.92 ± 0.15	1.09 ± 0.14	1.11 ± 0.15
Grants and Auxiliary	1.48 ± 0.2	1.54 ± 0.26	1.83 ± 0.37	1.95 ± 0.43
Total Expenditure	1.27 ± 0.13	1.29 ± 0.18	1.46 ± 0.22	1.48 ± 0.26
FSA Completions	1.09 ± 0.07	1.09 ± 0.09	1.09 ± 0.09	1.08 ± 0.11
First-time full-time	1.24 ± 0.06	1.28 ± 0.07	1.17 ± 0.04	1.16 ± 0.04
Total Faculty	1.16 ± 0.09	1.19 ± 0.11	1.18 ± 0.14	1.2 ± 0.16
Instructor Pay	1.2 ± 0.1	1.24 ± 0.13	1.4 ± 0.17	1.39 ± 0.19

eForm=

variable name	State Colleges	State Colleges UnGrouped	Community Colleges	Community Colleges UnGrouped
Student Tuition	0.99 ± 0.12	0.98 ± 0.13	0.85 ± 0.07	0.88 ± 0.08
Government Student Grant Aid	0.96 ± 0.05	0.95 ± 0.06	0.88 ± 0.02	0.88 ± 0.02
Government Appropriation	0.7 ± 0.06	0.67 ± 0.07	0.8 ± 0.03	0.77 ± 0.03
Government Grants and Contracts	0.65 ± 0.1	0.59 ± 0.11	0.71 ± 0.05	0.68 ± 0.05
Donation Investment	0.73 ± 0.18	0.71 ± 0.19	0.83 ± 0.1	0.81 ± 0.11
Auxiliary	0.7 ± 0.11	0.67 ± 0.12	0.75 ± 0.06	0.74 ± 0.06
Total Revenue	0.83 ± 0.05	0.81 ± 0.05	0.81 ± 0.02	0.8 ± 0.02
Instruction	0.9 ± 0.04	0.89 ± 0.04	0.81 ± 0.02	0.8 ± 0.02
Research	0.89 ± 0.27	0.82 ± 0.28	0.11 ± 0.3	0.16 ± 0.31
Public Service	0.9 ± 0.22	0.87 ± 0.24	0.71 ± 0.14	0.68 ± 0.15
Maintenance	0.8 ± 0.05	0.79 ± 0.05	0.88 ± 0.02	0.87 ± 0.03
Student Service	0.78 ± 0.05	0.77 ± 0.06	0.86 ± 0.03	0.86 ± 0.03
Grants and Auxiliary	0.73 ± 0.07	0.71 ± 0.07	0.93 ± 0.05	0.92 ± 0.05
Total Expenditure	0.82 ± 0.05	0.8 ± 0.05	0.83 ± 0.02	0.82 ± 0.02
FSA Completions	1.11 ± 0.05	1.11 ± 0.05	1. ± 0.03	0.99 ± 0.03
First-time full-time	1.11 ± 0.04	1.1 ± 0.04	0.79 ± 0.04	0.77 ± 0.04
Total Faculty	0.88 ± 0.04	0.86 ± 0.04	0.84 ± 0.02	0.84 ± 0.02
Instructor Pay	0.91 ± 0.04	0.89 ± 0.04	0.82 ± 0.02	0.81 ± 0.02

leForm=

variable name	Non-profit Private Colleges	Private non-profit colleges UnGrouped	Professional Schools	Professional Schools UnGrouped	For-profit Colleges	For-profit colleges UnGrouped
Student Tuition	1.17 ± 0.03	1.18 ± 0.03	1.11 ± 0.03	1.11 ± 0.04	0.99 ± 0.03	1. ± 0.04
Government Student Grant Aid	0.98 ± 0.04	0.98 ± 0.04	0.99 ± 0.02	0.98 ± 0.02	0.97 ± 0.05	0.98 ± 0.06
Government Appropriation	0.45 ± 0.24	0.4 ± 0.26				
Government Grants and Contracts	0.85 ± 0.08	0.87 ± 0.08				
Donation Investment	0.82 ± 0.06	0.85 ± 0.06	0.98 ± 0.16	0.92 ± 0.19	0.96 ± 0.21	0.89 ± 0.24
Auxiliary	1.18 ± 0.06	1.21 ± 0.06	0.51 ± 0.07	0.44 ± 0.08	0.93 ± 0.14	0.97 ± 0.15
Total Revenue	0.99 ± 0.03	1. ± 0.03	1.03 ± 0.02	1.03 ± 0.03	1. ± 0.03	1.01 ± 0.03
Instruction	0.99 ± 0.02	1. ± 0.03	0.97 ± 0.03	0.96 ± 0.03	0.93 ± 0.04	0.92 ± 0.05
Research	0.65 ± 0.14	0.64 ± 0.15				
Public Service	0.69 ± 0.14	0.68 ± 0.15				
Maintenance	0.89 ± 0.02	0.9 ± 0.03			0.75 ± 0.15	0.81 ± 0.16
Student Service	1.16 ± 0.03	1.17 ± 0.04	1.17 ± 0.04	1.18 ± 0.05	0.85 ± 0.07	0.83 ± 0.08
Grants and Auxiliary	1.05 ± 0.07	1.07 ± 0.08	0.71 ± 0.13	0.68 ± 0.15	1.2 ± 0.3	1.27 ± 0.34
Total Expenditure	0.95 ± 0.02	0.96 ± 0.02	1.06 ± 0.02	1.06 ± 0.03	0.87 ± 0.03	0.86 ± 0.03
FSA Completions	0.99 ± 0.04	1.02 ± 0.05	0.96 ± 0.06	0.93 ± 0.09	1.06 ± 0.09	1.01 ± 0.12
First-time full-time	1.09 ± 0.02	1.09 ± 0.02	1.02 ± 0.02	1.03 ± 0.02	0.96 ± 0.05	0.98 ± 0.06
Total Faculty	0.89 ± 0.02	0.89 ± 0.02	0.76 ± 0.02	0.72 ± 0.02	0.83 ± 0.04	0.8 ± 0.04
Instructor Pay	0.98 ± 0.02	0.99 ± 0.02			0.92 ± 0.24	1.02 ± 0.26

Table E-2 shows scaling results for all variables used in the main analysis, for all seven sectors, with and without delta grouped schools. Pairs of columns display main scaling results for complete sectors (which include Delta grouped schools) and "ungrouped" sectors that exclude grouped schools. Note that for-profit colleges' FSA completion and instructor pay results are discarded, the latter because of too few points.

The only sets of exponents that raise concern about Delta grouping are the following:

1) Removing Delta grouped schools shifts three scaling exponents enough to change their overall scaling behavior, because of their proximity to the somewhat arbitrary boundaries that we use to define linear scaling, from 0.95 to 1.05. However, each of these changes is slight and occurs well within the original exponent's confidence intervals. The changes do not alter our comparisons of scaling relationships within or between sectors, in the main text.
   a) Maintenance expenditure for public research universities changes from 1.07 ± 0.18 to 1.04 ± 0.14
   b) Grants and auxiliary expenditures for non-profit private colleges changes from 1.05 ± 0.07 to 1.07 ± 0.08
   c) Auxiliary revenues for for-profit colleges changes from 0.93 ± 0.14 to 0.97 ± 0.15

2) Tuition scaling for public research universities changes from 1.25 ± 0.13 to 1.34 ± 0.16 when grouped universities are excluded (see Figure E-1). However, this significant change to the exponent does not bring into question the superlinearity of the scaling relationship, nor does it affect how tuition scaling compares to the scaling of other salient university characteristics.

Figure E-1

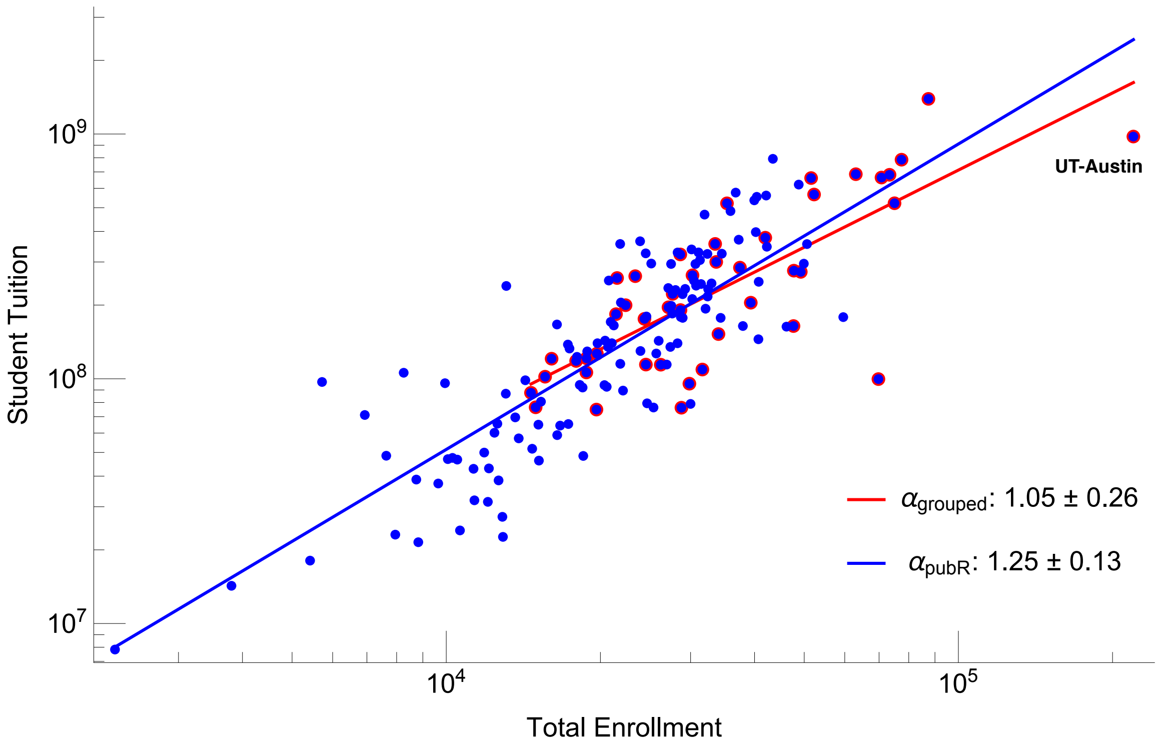

Figure E-1 shows that UT-Austin as one of the grouped public research universities drifted systematically downward from the fitline. $\alpha_{grouped}$ and α_{pubR} refer to grouped public research schools that have more than one campus and all public research schools respectively.

3) Total Faculty scaling for professional schools changes from 0.76 ± 0.02 to 0.72 ± 0.02 when grouped universities are excluded (see Figure E-2). Though the exponents' confidence intervals do not overlap, both are clearly sublinear, and the change does not affect how faculty scaling compares to the scaling of other salient university characteristics.

Figure E-2

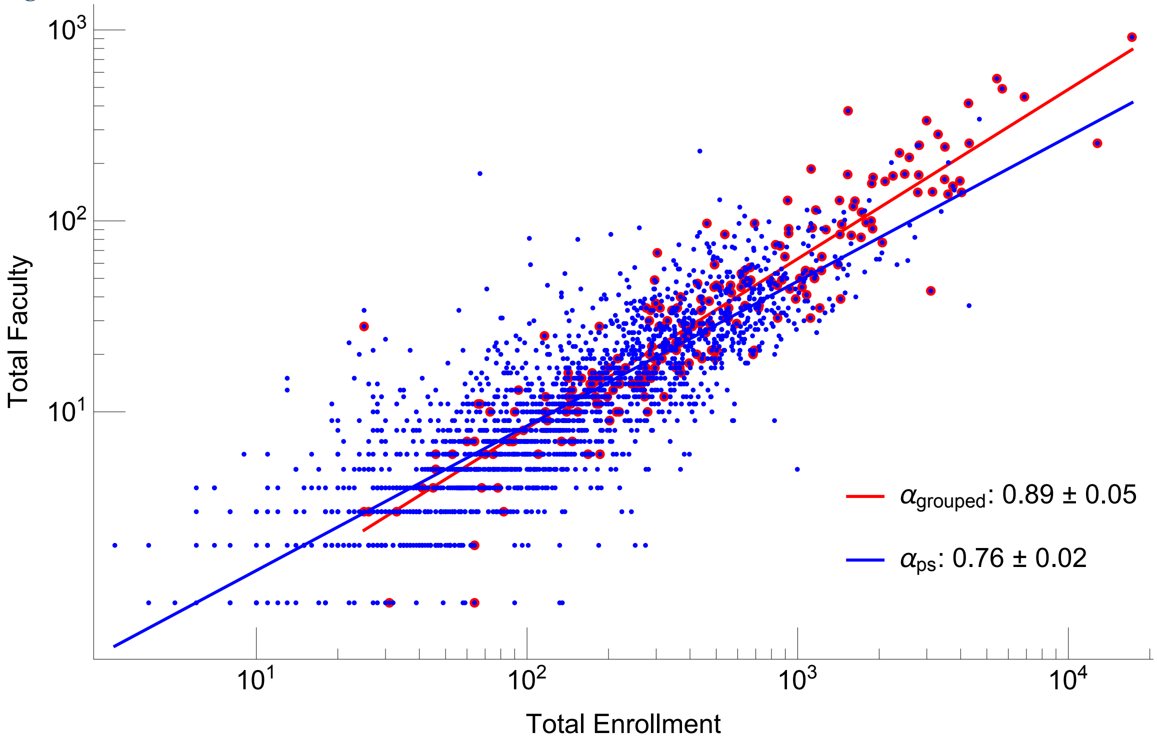

Figure E-2 shows that grouped professional schools drifted away from the overall fitline, toward linear scaling. Exponent values $\alpha_{grouped}$ and α_{ps} refer to grouped professional schools that have more than one campus and to all professional schools, respectively.

In the last two instances, as shown in Figure E-1 and Figure E-2, the scaling of grouped schools is closer to linear than the scaling of the entire sector (which includes both grouped and ungrouped schools). This effect can be explained mathematically and is to be expected. If we let x_i and y_i be the minimum values for size and an associated feature of a university respectively, then for a school at the maximum size (x_m, y_m) the largest possible error is given by grouping $N = x_m/x_i$ schools into one campus. In this case, for the fitted scaling relationship $y = y_0 x^\alpha$, the relative error at the maximum size is given by

$$\frac{(Ny_i - y_m)}{y_m} = \frac{(Ny_0 x_i^\alpha - y_0 x_m^\alpha)}{y_0 x_m^\alpha} = \frac{(x_m y_0 x_i^{\alpha-1} - y_0 x_m^\alpha)}{y_0 x_m^\alpha} = \left(\frac{x_i}{x_m}\right)^{\alpha-1} - 1$$

which will overestimate the general scaling if $\alpha < 1$ and underestimate the scaling if $\alpha > 1$. For example, if $y_m = 30y_i$ and $\alpha = 0.80$, then the relative error will be roughly 100%, and if $y_m = 1000y_i$ and $\alpha = 0.80$, then the relative error will be roughly 300%. We observe that the grouped campuses fall above the scaling relationship when $\alpha < 1$ in Figure E-2, and fall below the scaling relationships for $\alpha > 1$ in Figure E-1. More generally, Table E-2 shows that removing

these grouped schools typically raises superlinear exponents and lowers sublinear exponents, in line with the reasoning given here. However, it impacts each scaling relationship differently, and as we have emphasized, only very slightly.

This resilience of scaling relationships to removal of grouped institutions is an interesting result in its own right, and merits further investigation. The simplest explanation is that grouped schools are so few, and collapse together so few campuses, that they hardly impact the regression. We also think it is possible that for certain variables and sectors, grouped schools may actually follow the nonlinear scaling of the ungrouped schools, counter to their tendency towards linearity explained above. For example, grouped public research universities may disproportionately include medical schools, which may keep up their scaling exponent at the level of ungrouped research universities. Perhaps some other source of variance within the grouped universities keeps them within the overall variance. The implications run deep. We would expect grouped schools to tend toward linear scaling, if indeed the observed economies of scale depend upon greater populations of students, faculty and staff interacting in one place—as we hypothesize throughout the main paper, and analogous to the elegant interaction model of urban scaling, advanced by Luis Bettencourt (2013). The fact that we do not observe this strongly and uniformly across each sector, barring other explanations, may call into question our very notion of an institution. With more clearly disaggregated data, perhaps directly sourced from IPEDS, and better-defined levels of aggregation (separateness of campuses, student bodies, administrations, finances, government oversight, etc.), we could study how distributed institutional structure affects their scaling in general. We project that number and size of affiliate campuses could affect bureaucratic scaling differently from that of core functions, e.g. instruction and research, and that these very institutional economies of scale make it advantageous for universities to operate multiple campuses. Such research could shed light on the tradeoffs to institutional interconnectedness, decentralization, and complexity.

We conclude that grouping of schools does not affect our scaling results in general, and we continue to include Delta grouped universities in all sectors.

Delta grouping and matching with FSA Scorecard data

Delta grouping of universities raises a matching problem when using Delta data in combination with any other data on U.S. universities. The desired dataset may contain individual campuses or institutions that Delta instead groups together, so when simply merging this dataset to Delta data, only its data on the parent institution is appended to each Delta grouped university, and all its data on corresponding child institutions is omitted.

This institutional matching issue applies in every instance that we merge a dataset to Delta: Scorecard for FSA completions data, Chetty Mobility Report Cards for earnings data, Brookings for test score data, and HERD for research expenditure data. In each instance, we test whether it affects our results by removing all Delta-grouped institutions from the combined dataset, as described in the previous section, thereby narrowing the sample of universities in every sector to Delta "ungrouped" schools. These are single-campus schools that have never reported data to IPEDS together with another institution.

It is particularly important to examine how matching between Delta and Scorecard (explained in Appendix A) impacts our scaling analyses of FSA completions, because in our main scaling results by sector, we feature FSA completions alongside various revenue, expenditure, and faculty variables—all of which come from Delta data, as does total enrollment.

We compared results with and without Delta grouped schools for FSA completions versus FSA cohort in Table E-3, and FSA cohort versus total enrollment in Table E-4.

Table E-3

sector name	Grouped Schools	Ungrouped Schools	All Schools
Public Research Universities	1.12 ± 0.11; N:44	1.09 ± 0.09; N:115	1.09 ± 0.07; N:159
Private Research Universities	1.09 ± 0.15; N:20	1.09 ± 0.1; N:76	1.09 ± 0.08; N:96
State Colleges	1.16 ± 0.21; N:18	1.11 ± 0.05; N:335	1.11 ± 0.05; N:353
Community Colleges	1.01 ± 0.08; N:98	0.99 ± 0.03; N:644	0.99 ± 0.03; N:742
Non-profit Private Colleges	0.89 ± 0.09; N:62	1.01 ± 0.05; N:575	0.99 ± 0.04; N:637
Professional Schools	0.99 ± 0.06; N:88	0.95 ± 0.04; N:465	0.95 ± 0.03; N:553
For-profit Colleges	0.91 ± 0.04; N:51	0.93 ± 0.01; N:445	0.93 ± 0.01; N:496

Table E-3 shows the FSA completion vs. FSA cohort for grouped schools, ungrouped schools and all schools by sector. Neither variable comes from Delta. We observe that FSA completion scaling is all but identical between Delta ungrouped schools and all Delta schools. These results appeared in Table E-2 but are worth reiterating in this section. Delta grouped schools themselves scale somewhat differently from FSA schools, but scaling exponent confidence intervals still overlap. Disregard results for professional schools and for-profit colleges, which we exclude (see Appendix G).

Table E-4

sector name	FSA vs. Enrollment_Grouped	FSA vs. Enrollment_Ungrouped	FSA vs. Enrollment_All
Public Research Universities	0.53 ± 0.24; N:44	0.81 ± 0.07; N:115	0.71 ± 0.08; N:159
Private Research Universities	0.55 ± 0.19; N:20	0.87 ± 0.15; N:81	0.8 ± 0.12; N:101
State Colleges	0.53 ± 0.26; N:18	0.9 ± 0.04; N:356	0.86 ± 0.04; N:374
Community Colleges	0.49 ± 0.11; N:100	0.79 ± 0.03; N:787	0.75 ± 0.03; N:887
Non-profit Private Colleges	0.86 ± 0.15; N:75	0.88 ± 0.03; N:1048	0.89 ± 0.03; N:1123
Professional Schools	0.9 ± 0.08; N:200	1.02 ± 0.05; N:1543	0.96 ± 0.05; N:1743
For-profit Colleges	0.98 ± 0.54; N:58	0.18 ± 0.15; N:508	0.24 ± 0.14; N:566

Table E-4 shows FSA cohort vs. total enrollment for grouped schools, ungrouped schools and all schools by sector. Results vary more between Delta ungrouped schools and all Delta schools than they do for completions in Table E-3, but confidence intervals still overlap in every sector. Disregard results for professional schools and for-profit colleges, which we exclude (see Appendix G).

The two kinds of Delta grouped universities, multiple-campus institutions and multiple-institution systems, match differently with FSA completions data. The majority, 441, are multiple-campus institutions. They actually match well with FSA completions data because Scorecard aggregates FSA data for all campuses of each Title-IV institution, as Appendix G describes in a dedicated section. On the other hand, the 247 multiple-institution systems that Delta groups together are problematic. Each has the total enrollment of all schools in the system

combined, but has the FSA completions and FSA cohort of only the parent institution. In the completions versus cohort relationships, each Delta grouped system is included as the parent institution. These points are valid but omit child institution data. However, in the cohort versus completions relationships, each Delta grouped system has an erroneously small FSA cohort, relative to its total enrollment, and would appear on a plot below and to the right of the fitline. This explains why FSA cohort vs total enrollment scaling exponents increase when grouped schools are removed, and why these results are much more sensitive to the removal of grouped schools than completion vs cohort scaling.

Grouping of universities from multiple sectors

The Delta Cost Project is prone to grouping Title-IV institutions from different sectors. Such entries take the institutional characteristics of the linchpin (parent) institution, including its name and sector. Take two examples: first, the University of Maine System. It includes at least six independent four-year institutions plus Central Maine Community College, all of which Delta lists under its namesake main campus, a small public research university in the town of Orono. Second, Delta collapses the entire CUNY (City University of New York) into the entry CUNY City College in the state college sector. It includes nineteen locations, including several community colleges, a law school and CUNY Graduate School and University Center (CUNY School of Medicine is not included, since it did not begin operations until 2016, three years after our data year). With 268,825 students, it is the largest school in our dataset.

We were not able to determine precisely how many of the 247 grouped Title-IV systems this affected, but we estimate the number is low from scanning Parent Child Master List (https://deltacostproject.org/sites/default/files/ database/fy2015_parent-child-master-list.xls).

Delta acknowledges that its method of assigning the linchpin institution, and thereby the sector of the grouped university, is sometimes unclear (Delta Cost Project 2011, Jaquette and Parra 2016). We observe from their Parent Child Master List that Delta generally designates as the parent of a system one of its institution with the highest educational level. In other words, Delta incorporates two-year colleges into four-year colleges, and incorporates both two- and four-year colleges into research universities. We are confident that grouping these institutions in this way is no different from the much more commonplace Delta grouping of multiple campus institutions, where complicated combinations of institutional types effectively round up schools/campuses that would be in different sectors. In other words, we suppose that grouping the University of Maine System is akin to grouping schools such as the University of New Mexico and Kent State University in Ohio, which we justified earlier in this appendix. Therefore, we feel confident that grouping schools into a sector with a higher educational level does not significantly affect our results.

Grouping across sectors is problematic when it places schools in a different educational level than they belong. The CUNY system is one such example. CUNY groups not only community colleges but also graduate institutions under a state college (non-research public 4yr+) parent. On closer examination we find that CUNY reported awarding 463 doctoral degrees, far more than the twenty needed to qualify it as a research university according to the Carnegie Classification

(Carnegie Foundation for the Advancement of Teaching, 2011). Additionally, it reports spending $132,065,820 on research (roughly 3% of total expenditure) in 2013.

Appendix F: Revenue and expenditure component analyses

In this section we explain (1) the method by which we constructed the bar charts shown in Figure 3 of the main text and (2) how to interpret them with potential caveats in mind.

Constructing and reading the component bar charts

In the bar charts (see Figure F-1 as an example), each colored band corresponds to the per student amount of a given revenue or expenditure component, at a particular size bin along on the horizontal axis of the logarithm of total enrollment. Thus, as enrollment grows from left to right, a widening band indicates increase per person or superlinear scaling of that variable, while a narrowing band would indicate diminishing returns and sublinear scaling. For instance, Figure F-1 shows that student tuition revenue scales superlinearly at public research universities, from about $5,000 on average per student at a typical university of 10,000 students, up to about $9,000 at enrollments around 100,000.

The width of each band, representing the value of a revenue or expenditure variable at that size, is calculated from the fitted scaling equation for that particular component using the bin size. Bin sizes are evenly spread out with an arbitrary step size of $10^{0.5}$ within the actual range of enrollment. Only bins that have more than five schools and components that have more than 15 data points are shown in the final visualization.

Figure F-1

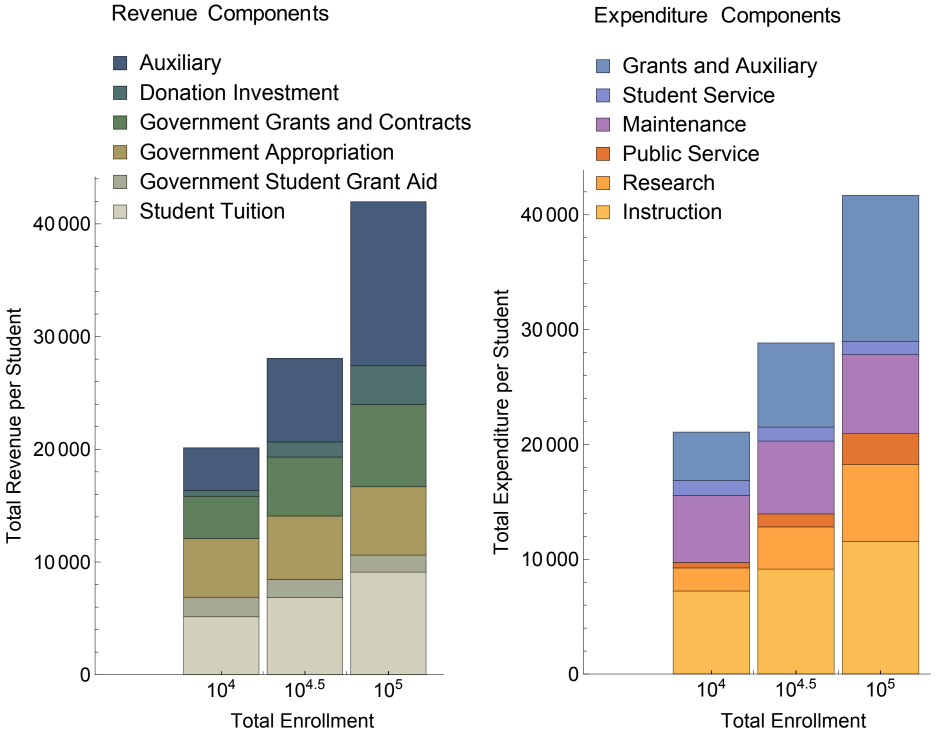

Figure F-1 shows both the total revenue and total expenditure component bar charts for public research universities.

In revenue component bar chart, from bottom to top, revenue bands include student tuition, government student grant aid, and government appropriation, all unrestricted but ostensibly dedicated toward covering educational and institutional costs; government grants and contracts dedicated toward research; unrestricted private funding, including donations, endowment and investment returns; and auxiliary revenues, restricted to auxiliary expenditures. Table B-1 of Appendix B defines each variable in detail.

In the expenditure component bar chart, up to six expenditure component variables are shown if data is available. The bottom three bands—instruction, research, and public service, in order of bottom to top—represent direct expenditures on three productive institutional functions, thereby outlining the scope of activities in each. The maintenance component above generally (though not exclusively) capture the various operational or administrative costs. The top two bands, student service and grants and auxiliary, represent costs that are less relevant to university functions. Noting that we group grants and auxiliary into one variable in expenditure component bar chart as grants contain a very small portion of the total expenditure and induce unnecessary granularity in visualization.

Qualifications to the bar chart visualization

The overall height of the bars, as the summation of component regression fitlines, corresponds roughly to the overall scaling behavior of total expenditure or revenue—increasing height indicates superlinearity whereas decreasing height indicates the opposite. However, the exponent

of the summation does not perfectly match up with the scaling of total variable. While the aggregated fitline describes the data with higher resolution, it may overweigh components which have the fewest data points. For example, most professional schools do not have grants and auxiliary expenditure, but the aggregated fitline treats all professional schools as having such spending, in amounts predicted by the few schools that do. Consequently, the expenditure bar chart in Figure F-2 slopes downward, indicating a diminishing return, and the corresponding summation of component fitlines, α_{sum} in Figure F-3, scales less than linearly in the same way. This is misleading: α_e, the scaling exponent of total expenditure versus total enrollment also in Figure F-3, has an exponent of 1.06 ± 0.02 (For-profit 2yr and 2yr- sectors both have linear scaling for total expenditure. When the sectors were combined into the sector professional schools, the exponent became barely superlinear. See Appendix D). This instance is the only mismatch we found between exponent values derived from summation and data. Conversely, all other bar charts, like the revenue component bar chart for professional schools (below in Figure F-2), give an accurate picture of overall scaling, where non-monotonic slope suggests linearity; the component line plot in Figure F-4 shows there is no substantive difference between α_{sum} and α_r for professional schools. Nonetheless, throughout the rest of the project, we only use actual scaling results derived directly from regression on total revenue and expenditure data to make statements about overall financial throughput in a given sector.

Figure F-2

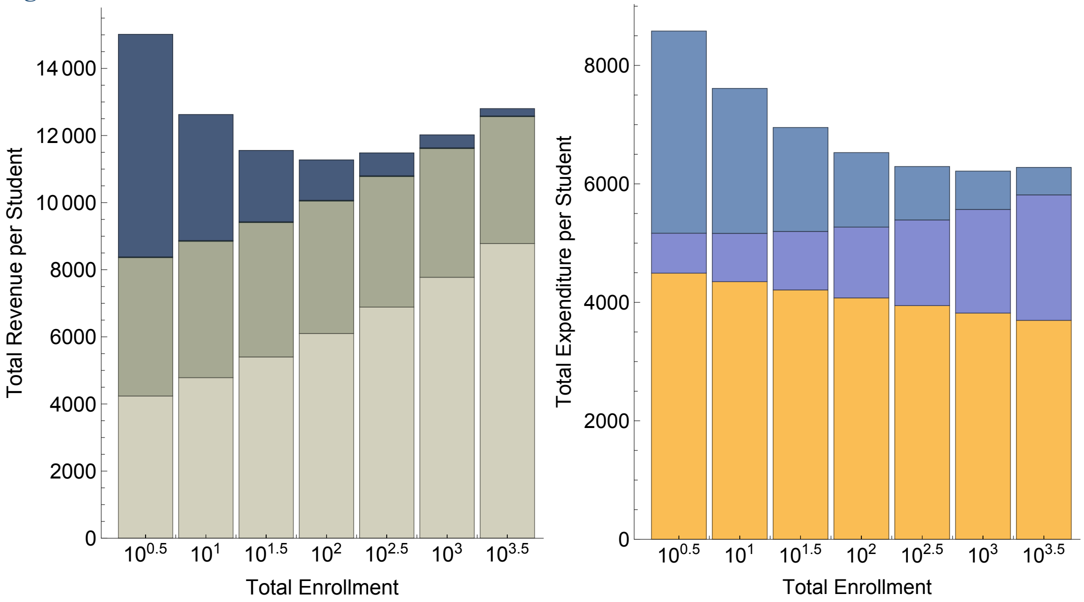

Figure F-2 shows the total revenue and total expenditure bar charts for professional schools. The bar heights of the expenditure bar charts misleadingly indicate sublinear total expenditure scaling because of the idiosyncratic summation of component regression lines.

Figure F-3

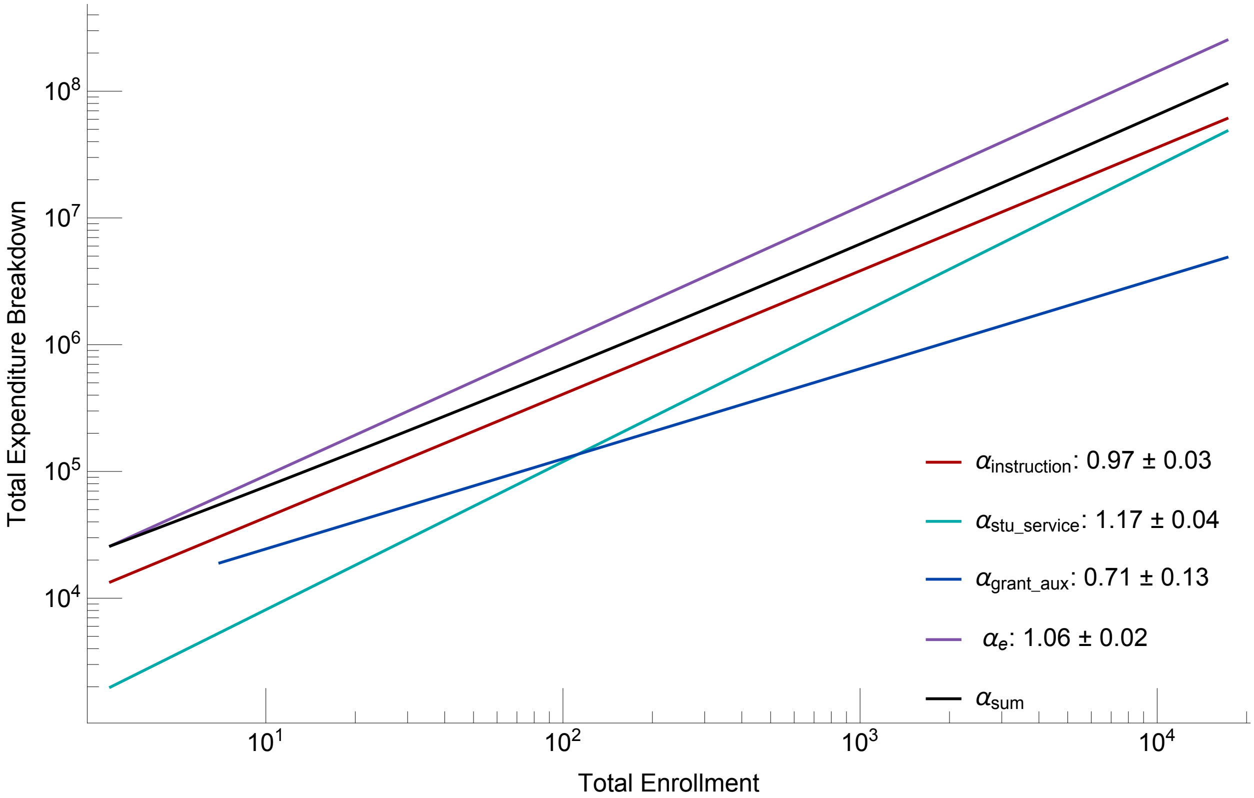

Figure F-3 shows the scaling of components of expenditure that have more than 15 data points for professional schools. The black line is calculated by adding up the three component fitlines, so by construction it is nonlinear and exhibits slight curvature; α_{sum} changes as a function of enrollment. Nonetheless we can see it is noticeably more sublinear than α_e , the scaling exponent of total expenditure. A significant gap opens up between the two lines at middle and large scales.

Figure F-4

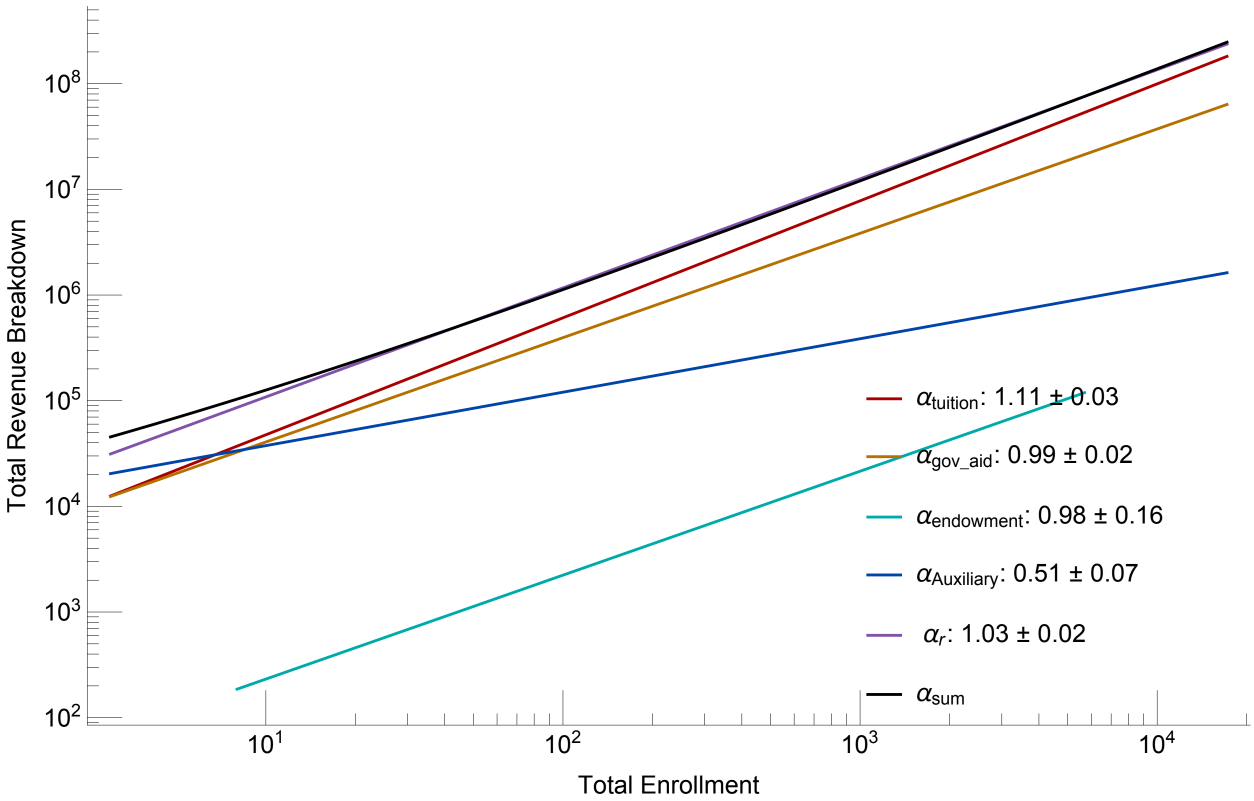

Figure F-4 shows the scaling of components of revenue that have more than 15 data points for professional schools. In this case, α_{sum}, which is calculated by adding up all component fitlines, scales extremely close to α_r, the scaling exponent of total revenue except at very small sizes. (Minor note: There are four components shown in the line graph, but only three components seem to appear in the revenue bar chart in Figure F-2. This is because endowment revenue in this sector is extremely small, about $23 per capita, so it becomes almost invisible in the bar chart though it is indeed there.)

Caveats regarding the data

First, we must point out the main disadvantage of condensing many scaling results conveniently into a single figure, as we do with each bar chart: to ensure readability, we omit precise scaling exponent values, confidence intervals, number of schools per size bin and all other descriptive statistics in order to ensure readability. The exponent values and confidence intervals for all these variables are provided in Appendix J.

Second, while IPEDS expense category definitions are precise, university reporting may not be reliable. For example, classifying a particular staff member under maintenance or student service is a judgment call that undoubtedly varies within and between universities. Such fuzzy reporting blurs the lines between purpose areas, and limits our assessment of tradeoffs. For example, the IPEDS expenditure variable of academic services has an educational element, but we combine it with operation maintenance and institutional support expenditures into one variable, maintenance expenditures, as our best measure of bureaucracy. Importantly, there is also a grey area between

instruction and research expenditures. It is unclear under which category research universities report expenses that have both educational and research components, such as wages to graduate students. The magnitude and distribution of these potential reporting errors are unknown, but we presume that it does not affect the key analyses.

Third, bar charts for some sectors lack certain revenue and expenditure component variables. In most cases these absences are expected—non-research universities do not spend money on research or public service, nor do private universities typically receive government appropriations or grants and contracts. The only exception is the maintenance expenditures from professional schools which have 17 data points that barely cross our threshold for regression.

Finally, while IPEDS has a well-defined category for R&D expenditure, it does not have a very satisfactory category for R&D revenue (i.e. funds raised by the university specifically for and as a result of its research activities). In the paper, we used a proxy, namely the amount of government grants (federal, state and local) that are for specific programs, research and projects, and excluding Pell grants. This is of course an imperfect measure. To gauge its reliability we additional examine here data from the Higher-Education Research and Development Survey (HERD) carried out annually by the NSF (see Appendix A). Unfortunately, the alignment between this dataset and our other datasets is low because the NSF requests administrators to fill in the survey for single campuses, which means that the R&D revenue for larger universities present in Delta data, many of which include multiple campuses (see Appendix E), will be underestimated and will cause noise at the higher end of the scale. Additionally, given that this data is reported to the NSF, there may be biases in the reporting.

We find that R&D expenditure from HERD and R&D expenditure from Delta Cost are in somewhat good agreement, except at higher scales where the HERD data shows systematically lower expenditure (either because of the difference in the unit of analysis between the two datasets or because of inconsistent reporting to different institutions). R&D revenue from HERD are imperfectly correlated with the Government project grants and contracts data we use in our main analysis. Re-estimating the scaling relationship for R&D revenue (a measure of R&D output) using the HERD data, we find that it is extremely uncertain, showing scaling coefficients that are of the order of those shown in the paper but with much greater uncertainty (See Table F-1). We believe this is due to the difference in the unit of analysis between HERD and Delta data. It also points to the fact that better data is needed on research output to deepen our understanding of how institutional size affects the research output of a university.

Figure F-5

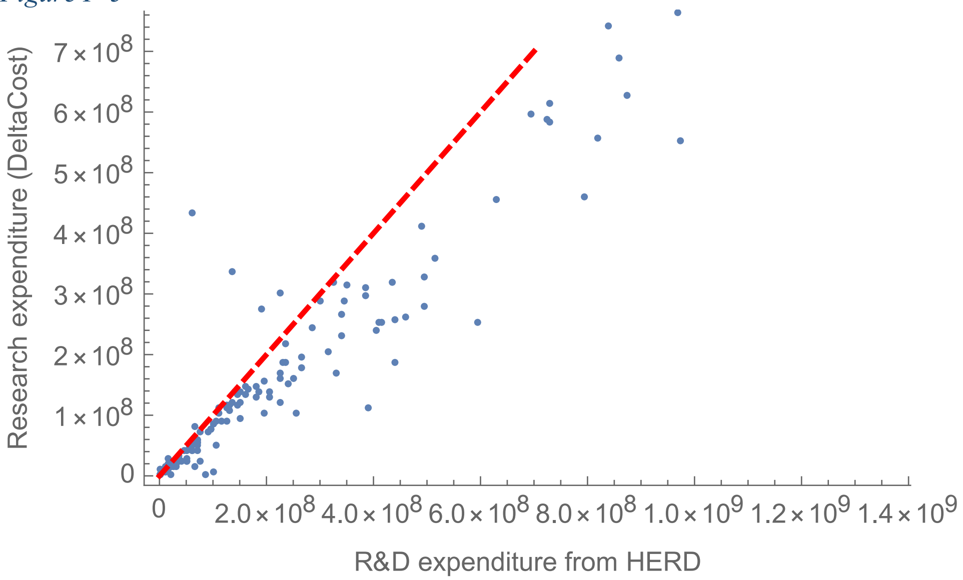

Figure F-5 shows the correlation between Research expenditure data from Delta Cost and NSF HERD data (for public research universities).

Figure F-6

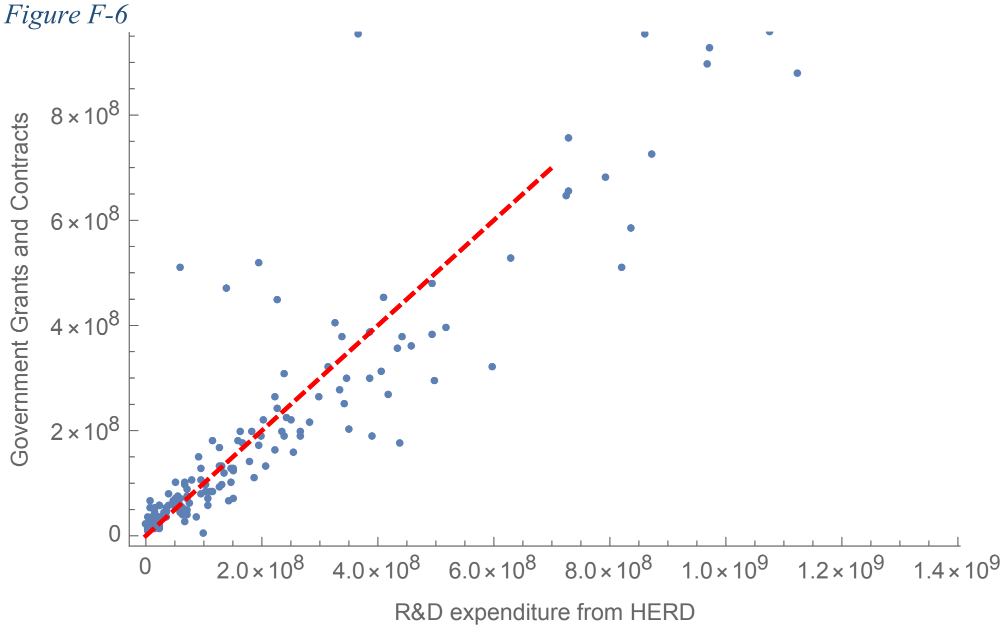

Figure F-6 shows the correlation between Government grants and contracts data from Delta Cost (proxy for research revenue used in the main analysis) and NSF HERD data on revenue (for public research universities).

Table F-1

sector name	R&D revenue from HERD	R&D expenditure from HERD
Public Research Universities	1.28 ± 0.31	1.3 ± 0.29
Private Research Universities	1.53 ± 0.77	1.46 ± 0.72
State Colleges	0.61 ± 0.24	0.61 ± 0.2
Community Colleges		
Non-profit Private Colleges	0.05 ± 0.21	0.07 ± 0.17
Professional Schools		
For-profit Colleges		

Appendix G: Completion rate analyses

To examine how postsecondary educational programs perform with scale, our analysis includes data on the scaling of total completions. We use the two best publicly available and most widely reported measures of completions in U.S. higher education to measure educational output in this most basic sense: completion rates of Federal Student Aid-receiving student cohorts (referred to as FSA completions) and completion rates for first-time full-time students (referred to as FTFT completions). In this Appendix, we explain (1) how we compose the two total completion metrics from raw data; (2) why we use FSA and FTFT cohorts as the size variables for our completion scaling analyses, how these cohorts relate to total enrollment, and why we favor FSA results over FTFT results; and (3) special cleaning we employed to the data to avoid the impacts of a campus grouping problem with FSA data (separate from the Delta grouping problem explained in Appendix E).

Total Completions Metrics for FSA and FTFT cohorts

National Student Loan Data System (NSLDS) tracks every Federal Student Aid (FSA) receiving student through their entire postsecondary career. The data used here capture the 2013 educational outcomes of federal student loan receiving students whose first aid year was 2007 (grant receiving students were not tracked until 2012). We use FSA Cohort (6yr), the population of that cohort, instead of total enrollment as the relevant measure of size for the scaling of this outcome variable. Table G-1 shows how FSA and FTFT cohorts are defined.

Each university reports a complete spectrum of all possible student outcomes for its FSA cohort. These outcomes include the percentage of FSA students who graduated within six years from the original institution, from a different four-year institution, and from a different two-year institution. We group these percentages together to obtain the six-year completion rate at each institution of original enrollment. See pages 21-23 of College Scorecard Full Data Documentation (https://collegescorecard.ed.gov/assets/FullDataDocumentation.pdf) for full variable descriptions and Appendix B for more details. Note that because of privacy considerations, College Scorecard suppresses any datum with under 30 students, for any student outcome at any school. Some small schools are thus excluded from our completion rate analysis.

Total completion data for full-time first-time (FTFT) students is found in Delta data, which sources it from the IPEDS Graduation Rate Survey. For 4yr schools, we use the cohort that starts in 2007 to match with FSA cohorts. For 2yr and 2yr- universities, we have 3yr graduation rates

instead of 6yr graduation rates (in both cases, the data gives us completion rate within 150% of normal time to completion), hence for those schools we use 2010 starting-year cohort data.

Unlike FSA completion rate data, FTFT completion rate only includes students who graduate from the same institution. In the community college sector, transferring to a 4yr school is a common and favorable outcome, so we find FTFT completions less informative than FSA in this sector. In the professional school sector, none of the 1393 2yr- schools report graduation rate. The scaling result we use describes the 730 of 798 2yr colleges that do report 3yr FTFT graduation rate.

For the FTFT completion scaling results, this documented graduation rate for FTFT students is regressed versus the corresponding FTFT student cohort.

Table G-1

	Under-graduate only	Full-time	Part-time	Students who transferred in from a previous institution	Students who transferred away to a different institution	Federal Student Loan (FSL) (lower-income population)	Pell Grant only, no FSL	Non-aid-receiving
FSA	✓	✓	✓		✓	✓		
FTFT	✓	✓				✓	✓	✓

Table G-1 compares the definition of FSA and FTFT cohorts. Check marks indicate that the corresponding cohort includes *some but not necessarily all students* of the type given by the column.

FSA and FTFT cohorts versus total enrollment

Total enrollment, including full-time and part-time students or undergraduates and graduates, is the key size variable that all aforementioned institution-wide financial flows we analyzed were mapped on. However, we are limited by such comparison due to the lack of matched completion data for total enrollment. In Table 2 of the main text and throughout, we mix size variables when assessing each sectors' functional tradeoffs: we use completion cohort to assess educational output scaling (via completions), but total enrollment to assess scaling of affordability (via tuition) and all other variables / purposes. This crisscrossing of size variables limits to some extent our ability to accurately assess tradeoffs. We assume that the incongruence is small enough for our results to still be meaningful, and here examine the validity of this assumption.

What are the proportions of total enrollment that FSA and FTFT students constitute? Table G-2 indicates that FSA cohorts on average represent between 45% to 92% of one-year total enrollment across all sectors, while this share is between 24% to 26% for FTFT cohorts. In addition, more than 80% of schools have both FSA and FTFT cohort data in most sectors, supporting the usage of these two metrics to construct narratives of completion against revenue and expenditure.

Table G-2 also indicates that FSA and FTFT cohorts’ proportions of total enrollment change with scale. Typically, the proportion of FSA students decreases as total enrollment increases,

indicating that larger schools may have greater shares of wealthier students. Hence FSA cohorts become less representative and FSA completions lose some of their predictive power with scale. In contrast, FTFT cohorts grow more than proportionally versus total enrollment for research schools, telling us that FTFT completions may actually have greater confidence for larger research schools, as a measure of overall graduation rate and educational throughput. Consequently, understanding that time commitment and economic background of students are embedded differently in the stratified education sectors according to their missions, we can choose one metric over another depending on the size of schools and their unique student composition. For example, in the case of community college, we trust FSA rather than FTFT as this sector attracts many part-time and less wealthy students.

Overall, we give more weight to the results derived from FSA completions than FTFT completions because aid-receiving cohorts represent a larger proportion of the student body, may be more representative and the FSA completions includes the possibility that the student graduates at a transfer institution.

Table G-2

bleForm=

sector name	FSA vs. Total Enrollment	FTFT vs. Total Enrollment
Public Research Universities	0.71±0.08; AvgPct_FSA:0.58; N_FSA:159; N_Total:160	1.16±0.13; AvgPct_FTFT:0.3; N_FTFT:159; N_Total:160
Private Research Universities	0.82±0.12; AvgPct_FSA:0.45; N_FSA:99; N_Total:102	1.27±0.24; AvgPct_FTFT:0.33; N_FTFT:99; N_Total:102
State Colleges	0.86±0.04; AvgPct_FSA:0.78; N_FSA:374; N_Total:382	0.92±0.13; AvgPct_FTFT:0.24; N_FTFT:319; N_Total:382
Community Colleges	0.75±0.03; AvgPct_FSA:0.88; N_FSA:883; N_Total:908	0.44±0.04; AvgPct_FTFT:0.28; N_FTFT:889; N_Total:908
Non-profit Private Colleges	0.91±0.03; AvgPct_FSA:0.92; N_FSA:1099; N_Total:1373	1.06±0.05; AvgPct_FTFT:0.36; N_FTFT:1035; N_Total:1373
~~Professional Schools~~	~~0.85±0.04; AvgPct_FSA:1.69; N_FSA:1139; N_Total:2230~~	~~0.89±0.07; AvgPct_FTFT:0.82; N_FTFT:729; N_Total:2230~~
~~For-profit Colleges~~	~~0.84±0.11; AvgPct_FSA:1.47; N_FSA:146; N_Total:647~~	~~0.63±0.14; AvgPct_FTFT:0.06; N_FTFT:281; N_Total:647~~

Table G-2 shows the following statistics for each sector (from left to right): (1) the exponent of the scaling relationship regressing FSA or FTFT cohort versus total enrollment, (2) average percentage of FSA or FTFT students in schools that have FSA or FTFT, (3) number of schools that have FSA or FTFT data, and (4) total number of schools. Note that we discarded data from professional schools and for-profit colleges (discussed below).

FSA grouping problem and unusable FSA completions data

As discussed in Appendix E, the Delta Cost Project groups together campuses and schools whenever they have reported any IPEDS data together, such as their net assets in the IPEDS Finance Survey. However, not all multiple campus institutions have done so. Delta data includes separate entries for the 144 campuses of ITT Technical Institute, the 63 campuses of the University of Phoenix, and so on for other institutions—especially for-profit schools. We regard these schools as functionally separate institutions.

This becomes a problem when merging datasets. While Delta does not group these multiple campus institutions, College Scorecard always groups their FSA completion data, as noted in the data documentation:

> "While these [Title-IV institution] data are reported at the individual level to NSLDS and used to distribute federal aid, they are published only at the aggregate institutional

level. While some schools report these data at the campus level (8-digit OPE ID), we have rolled those up to the institution level (6-digit OPE ID)."

In practice, each campus of a so-called "FSA grouped" institution is still listed individually in College Scorecard, but in place of unique figures for completion rate and completion cohort, all campuses have identical, aggregated values. Thus, in our combined dataset, the 63 campuses of the University of Phoenix have unique total enrollments, ranging from 43 to 256,402 students, but they all have the same number of completions: 170,334.

Figure G-1

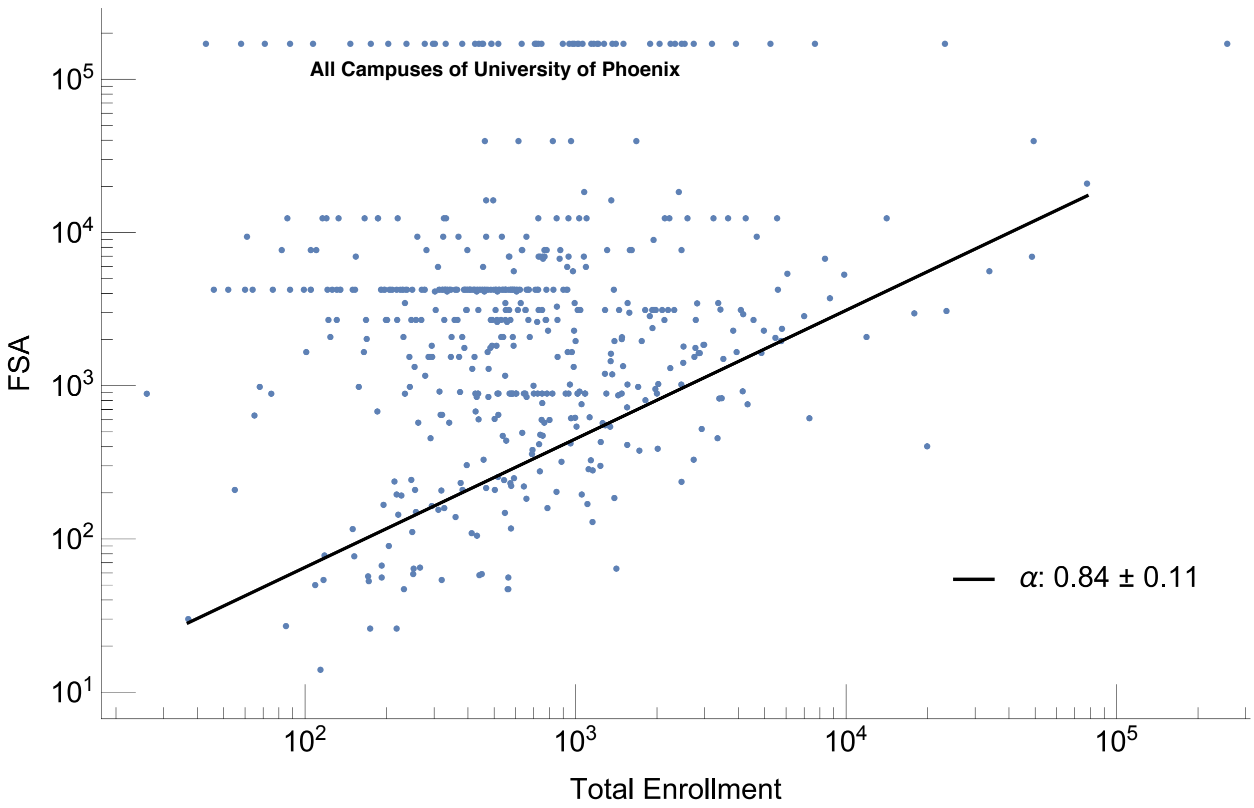

Figure G-1 shows FSA Cohort (Scorecard) versus Total Enrollment (Delta) for for-profit colleges, where the problem is most severe. Campuses with grouped FSA data appear as horizontal streaks, at the upper-left corner of the plot. When including these erroneous points, the regression fits terribly, and has a scaling exponent of 0.20 ± 0.13. The fitline was calculated by filtering out schools from our combined database with the same 6-digit OPEID and the same FSA Cohort.

Figure G-2

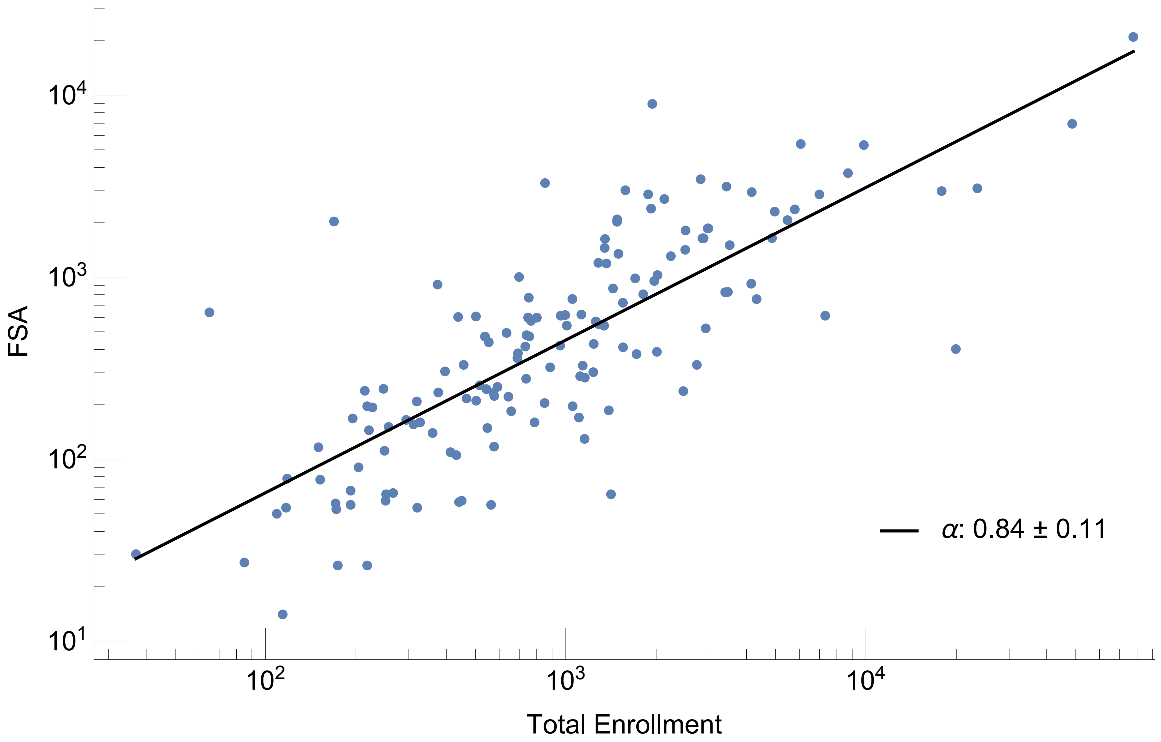

Figure G-2 shows the cleaned data by itself, for the for-profit colleges. While the streaks are gone, and it looks significantly better, the majority of points had to be removed, and there is still a problem. The FSA cohort of a university, or any component of its enrollment, should never exceed total enrollment. We should see a hard cut-off at the one-to-one line, as we do in public and private non-profit sectors. Instead, we observe several points far above the one-to-one line (not shown but easily visualized using the tick marks). We also observe this for Professional Schools. This leads us to believe that our method of removing schools with inappropriately grouped FSA completion data has failed in these sectors, and that their grouped FSA data could not be harmonized with Delta data's grouping. We conclude by discarding FSA completion data entirely for for-profit colleges and professional schools. Meanwhile we remove the few schools in other sectors with inappropriately grouped FSA completion data.

Table G-3

sector name	FSA Completion	FSA Completion Without Removal
Public Research Universities	1.09 ± 0.07	1.09 ± 0.07
Private Research Universities	1.09 ± 0.09	1.09 ± 0.08
State Colleges	1.11 ± 0.05	1.11 ± 0.05
Community Colleges	1. ± 0.03	0.99 ± 0.03
Non-profit Private Colleges	0.99 ± 0.04	0.99 ± 0.04
Professional Schools	0.96 ± 0.06	0.95 ± 0.03
For-profit Colleges	1.06 ± 0.09	0.93 ± 0.01

Table G-3 shows the scaling exponents for Total Completion vs. FSA cohorts with and without removing FSA grouped universities. Their removal visibly raises the scaling exponent for for-profit colleges—an important red flag—but not for professional schools or any other sector.

Nonetheless, we consider both for-profit and professional school FSA completion data invalid for the above reasons pertaining to FSA cohorts and total enrollment.

Appendix H: Tuition, selectivity and post-graduation earnings

We turn here to a few concerns and robustness tests relevant for Figure 4. The unit of analysis in the Equality of Opportunity dataset of earnings does not align perfectly with that of Scorecard because of the presence of some OPEID campuses being grouped under a single super-OPEID, as discussed in Appendix A. The alignment is poorer still with the Delta dataset since this dataset uses UNITIDs (with its own idiosyncratic groupings of campuses). Hence, we reproduced all the graphs, omitting any grouped campuses (whether super-OPEID or the groupings in Delta) to check that the qualitative patterns we discuss in the Discussion are robust to these data merging problems, finding that only a few observations are affected by this unit problem.

Another potential issue in Figure 4 is that we are using data from 2013, as in the rest of the paper. However, the earnings data displayed is for individuals born in 1984, who started college around 2002-2003. The relationship between these two variables is informative of the tradeoff between tuition and mid-career earnings that students in 2013-2014 might have considered by gauging mid-career earnings of young adults in their 30s. Yet, we may also want to know the relationship between earnings and the tuition paid by the students whose earnings we analyze. We thus reproduce the graphs here with tuition data from 2003. We see that out-of-state tuition (i.e. market price) was considerably less at the high-end (for non-profit private colleges and private research universities), while the distribution of the average net tuition has remained more constant. The relative position of sectors is similar to that in Figure 4 of the main text in the curve relating out-of-state tuition to mean earnings. When looking net tuition paid by students, we see that sectors were more starkly differentiated in the financial added value they provided in 2003 (second panel), and very clearly dominated by the public sector schools.

Figure H-1

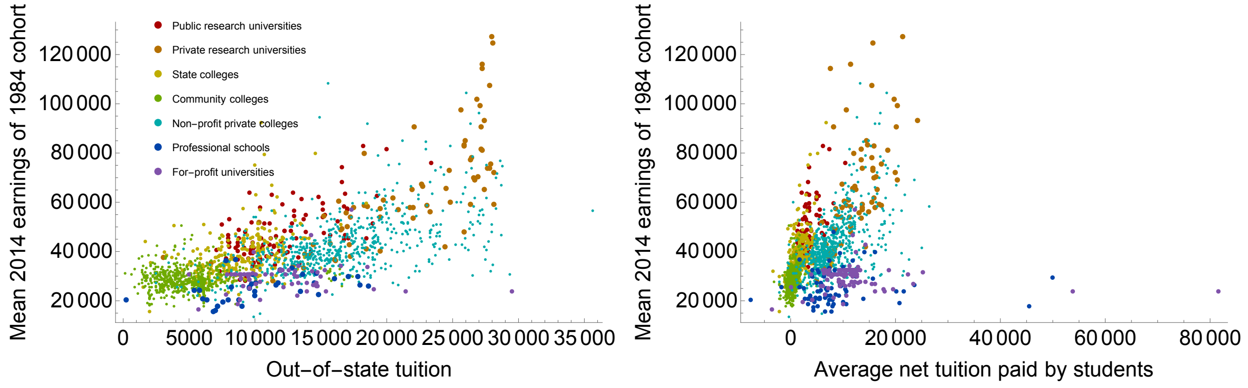

Figure H-1: Figure 4 with 2003 tuition data rather than 2013 tuition data. Average net tuition paid by students is the tuition variable used throughout the analysis, a component of revenue from Delta and the IPEDS Finance Survey.

Table H-1

sector name	Total 2014 earnings of 1984 cohort
Public Research Universities	0.85 ± 0.11
Private Research Universities	1.11 ± 0.15
State Colleges	0.97 ± 0.04
Community Colleges	0.92 ± 0.04
Non-profit Private Colleges	1.13 ± 0.05
Professional Schools	1.01 ± 0.09
For-profit Colleges	0.63 ± 0.1

Table H-1: Scaling of total earnings, using 2003 data for the size of universities.

Finally, part of the SAT scores data from Brookings are imputed for schools that are missing them, generally because they are fairly unselective. The additional imputed data makes it easier to discuss all sectors. However, the model for this imputation does include tuition and the Carnegie classification, so we reproduce the Figure in the main text here with raw score data from Scorecard. This covers fewer schools and almost entirely leaves out certain sectors, but shows that the common monotonic relationship between selectivity, tuition and earnings is apparent in the raw data, as well as the observation that non-profit private colleges are much more heterogeneous than other sectors.

Figure H-2

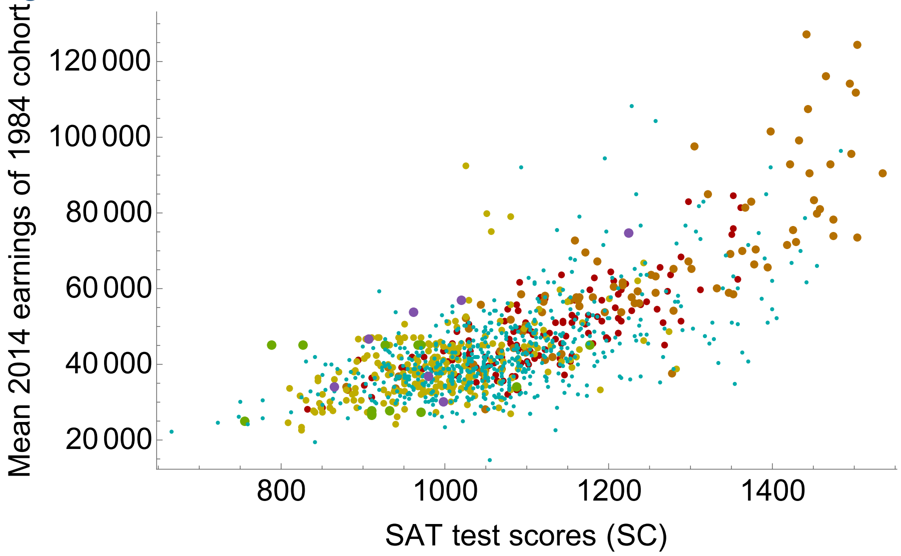

Figure H-2: Mean earnings versus raw (non-imputed) SAT test scores from the Scorecard dataset.

Appendix I: Overall magnitude analysis of higher education

An important consideration within our analysis beyond scaling behavior is the overall magnitude of properties. For example we are not only interested in whether certain types of efficiencies or returns scale more quickly in one sector than another, but also in the overall magnitude of these features. While it may be encouraging if some measure of return or efficiency scales more steeply with increasing size in one sector compared with another, our enthusiasm should be tempered if that sector's overall magnitude is consistently lower. Figure I-1 to Figure I-11 provide the absolute scale of all of the main scaling relationships of interest. These plots show,

for example, that the public and private research universities not only have steeper scaling relationships for instruction with total enrollment but also a larger overall magnitude for most enrollments.

Similarly, for considerations of scaling-up higher education we are interested in the scales at which certain sectors outcompete one another. These crossing points are implicit in the absolute magnitude analysis discussed above and in Figure I-1 to Figure I-11, and can be more easily seen by looking at the scaling relationships on a per capita basis. The overall of the per capita scaling relationships for all sectors are given in Figure I-12 to Figure I-14.

Figure I-1

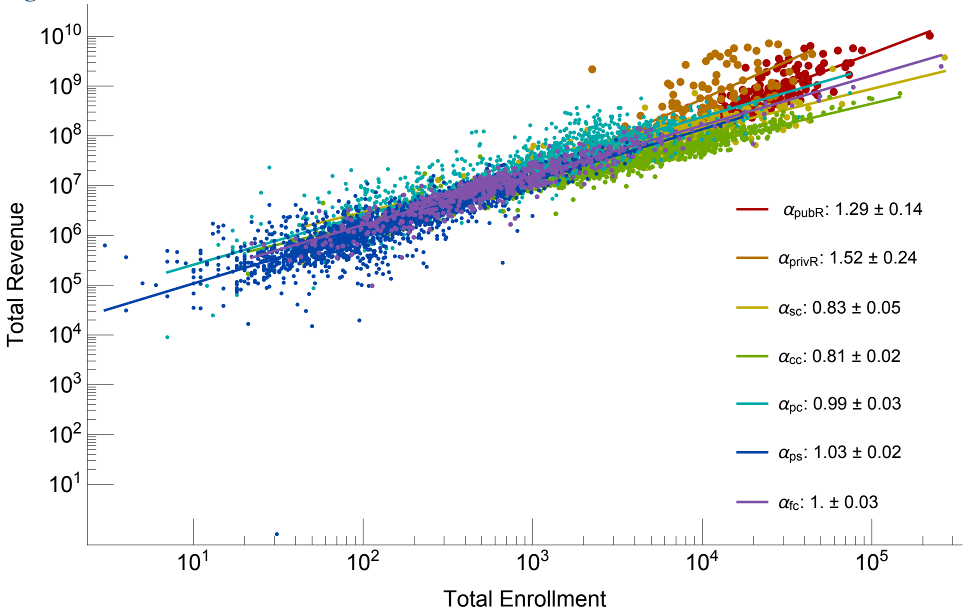

Figure I-1 shows fitlines and data for total revenue versus total enrollment, for all sectors.

Figure I-2

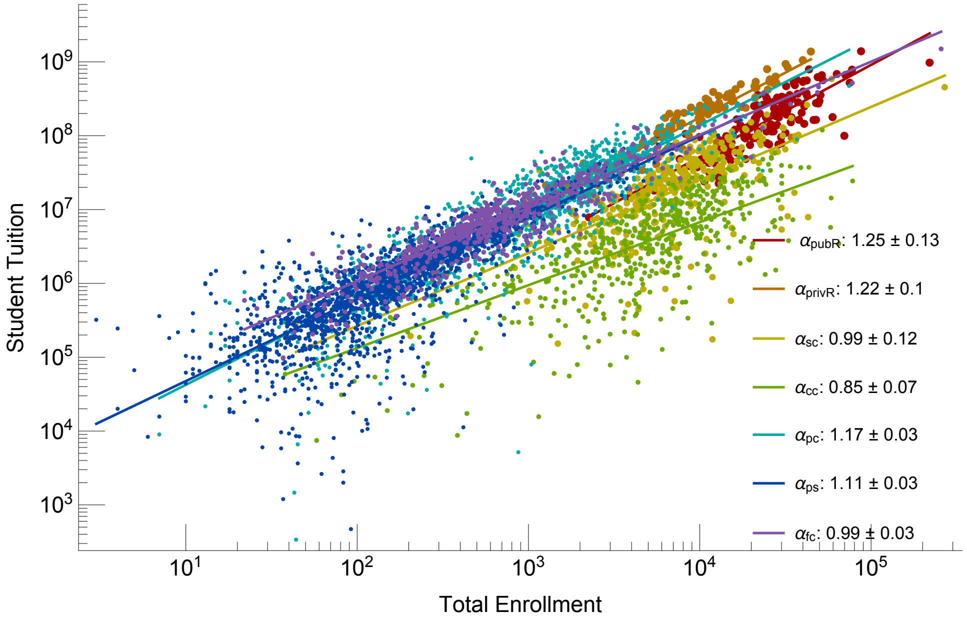

Figure I-2 shows fitlines and data for student tuition revenue versus total enrollment, for all sectors.

Figure I-3

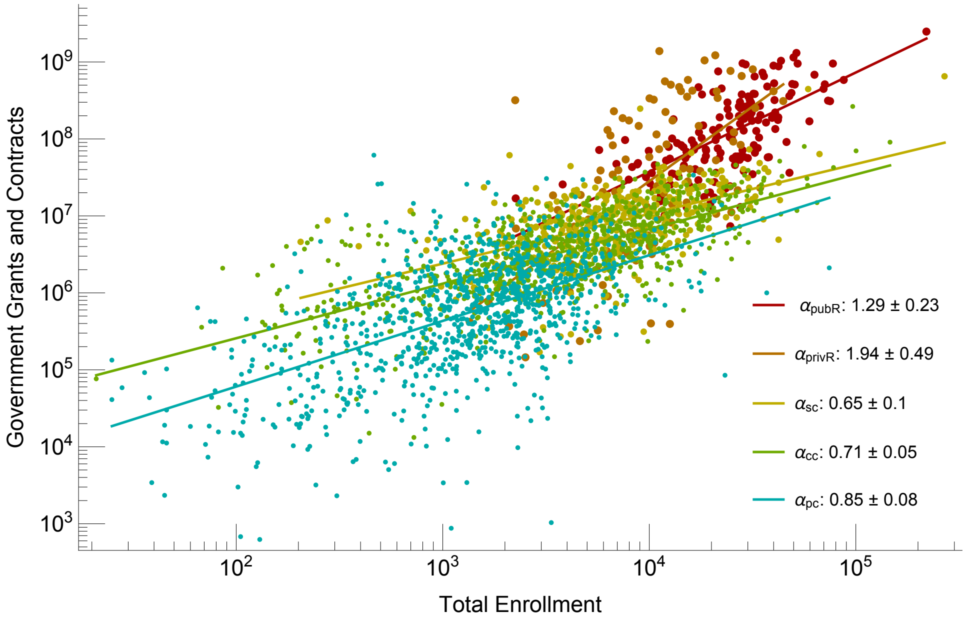

Figure I-3 shows fitlines and data for government grants and contracts (a proxy for research revenue) versus total enrollment, for all sectors.

Figure I-4

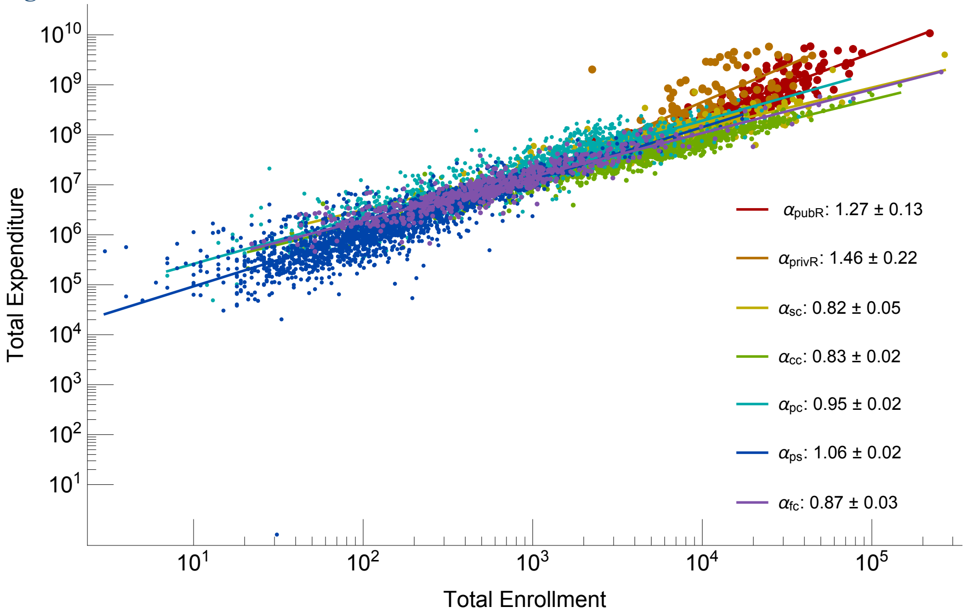

Figure I-4 shows fitlines and data for total expenditure versus total enrollment, for all sectors.

Figure I-5

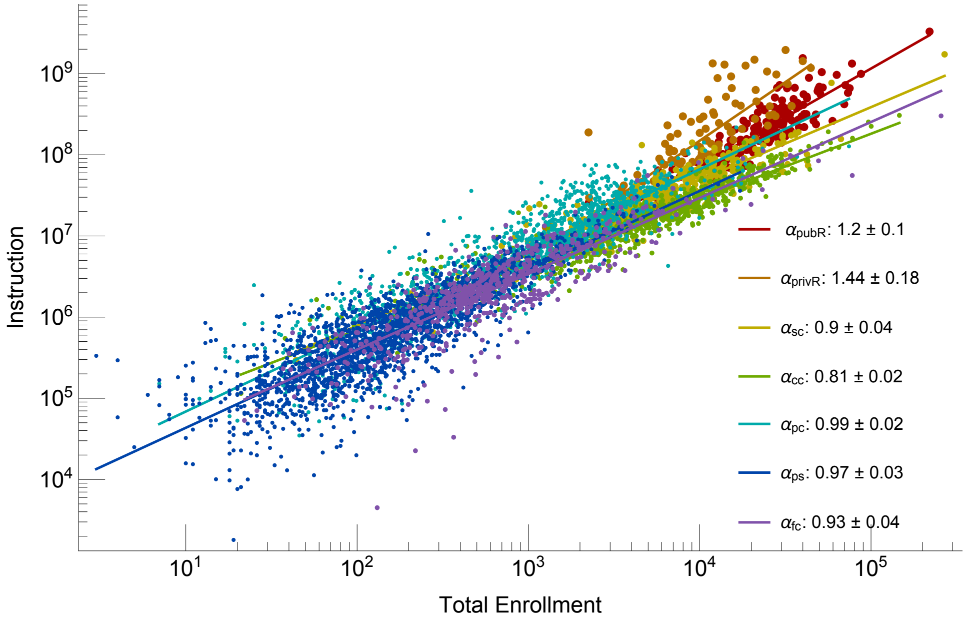

Figure I-5 shows fitlines and data for instruction expenditure versus total enrollment, for all sectors.

Figure I-6

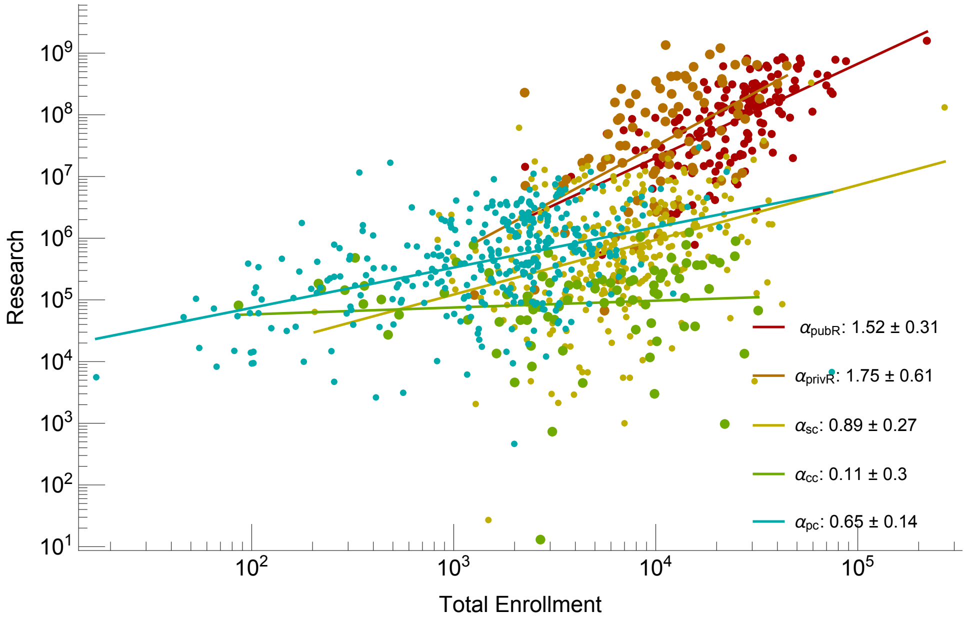

Figure I-6 shows fitlines and data for research expenditure versus total enrollment, for all sectors.

Figure I-7

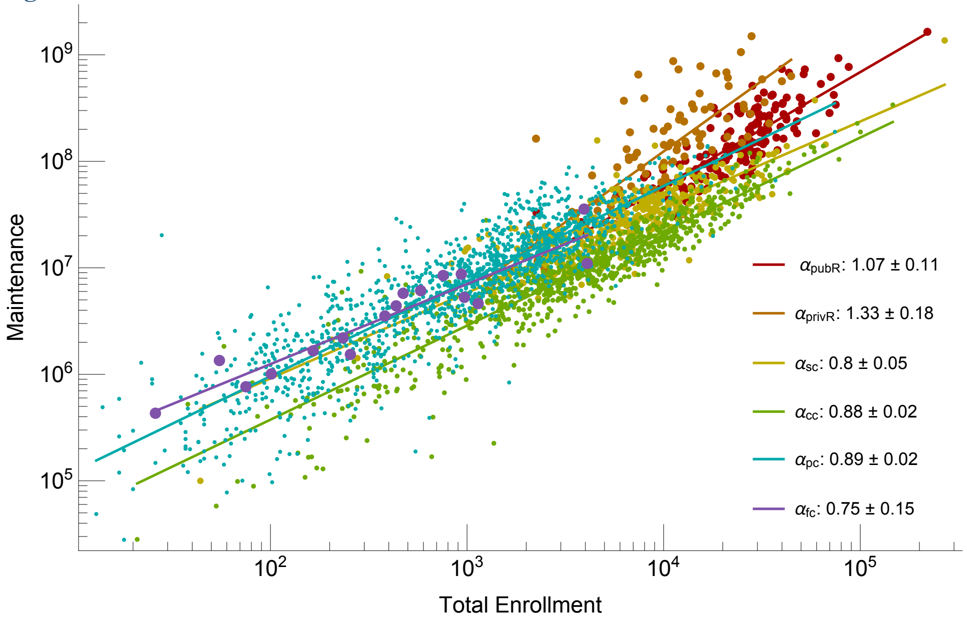

Figure I-7 shows fitlines and data for maintenance expenditure versus total enrollment, for all sectors.

Figure I-8

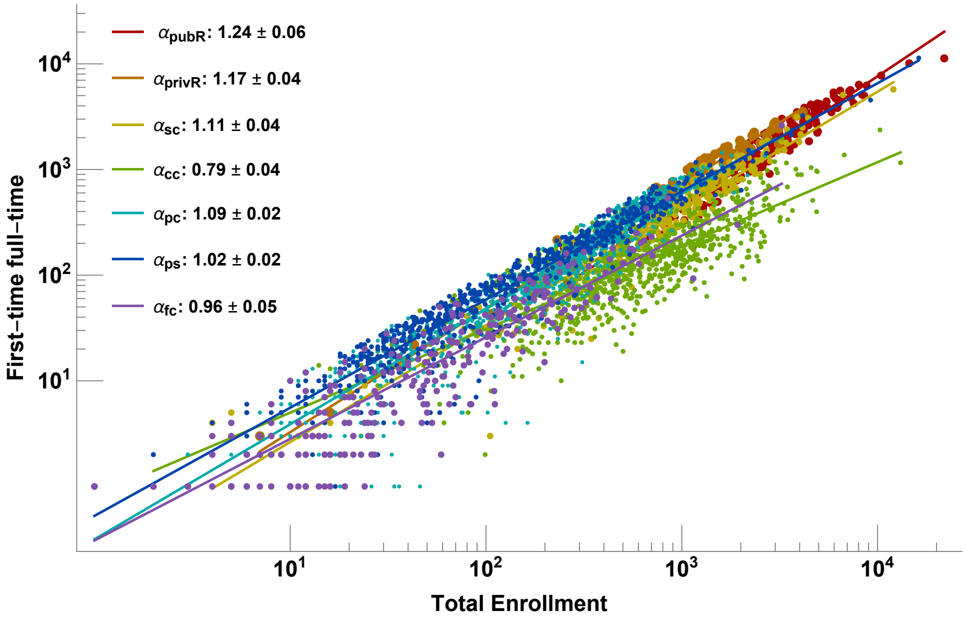

Figure I-8 shows fitlines and data for FTFT completions versus total enrollment, for all sectors. These scaling results are distinct from FTFT completions versus FTFT cohort.

Figure I-9

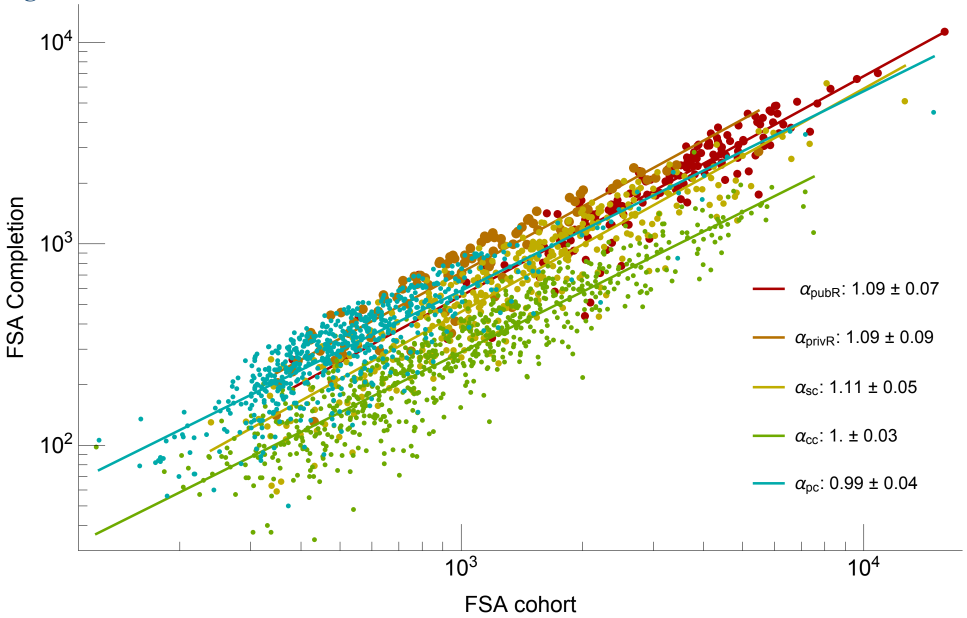

Figure I-9 shows fitlines and data for FSA completions versus FSA cohort, for all sectors.

Figure I-10

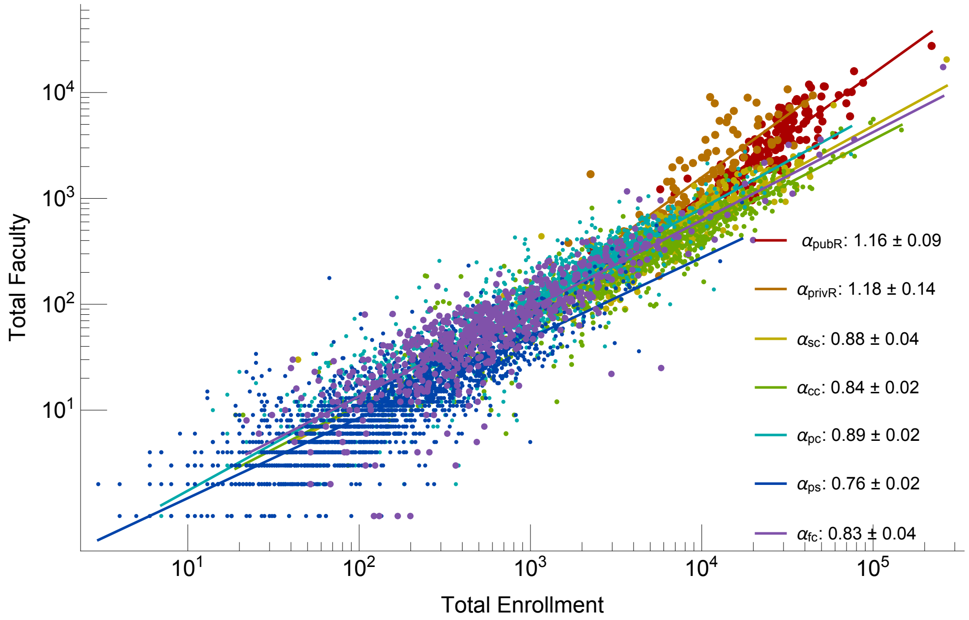

Figure I-10 shows fitlines and data for total faculty versus total enrollment, for all sectors.

Figure I-11

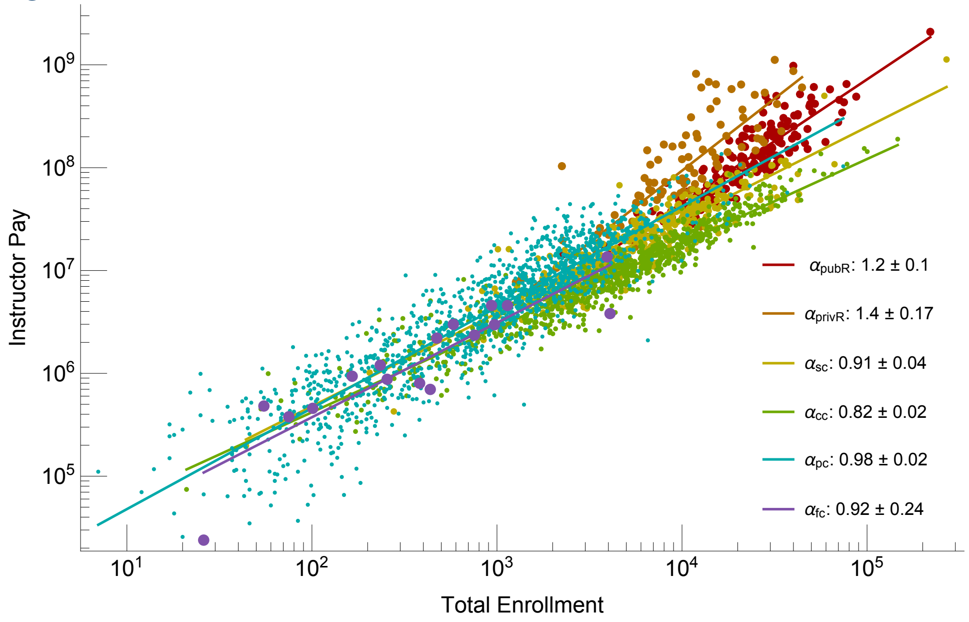

Figure I-11 shows fitlines and data for instructor pay versus total enrollment, for all sectors.

Figure I-12

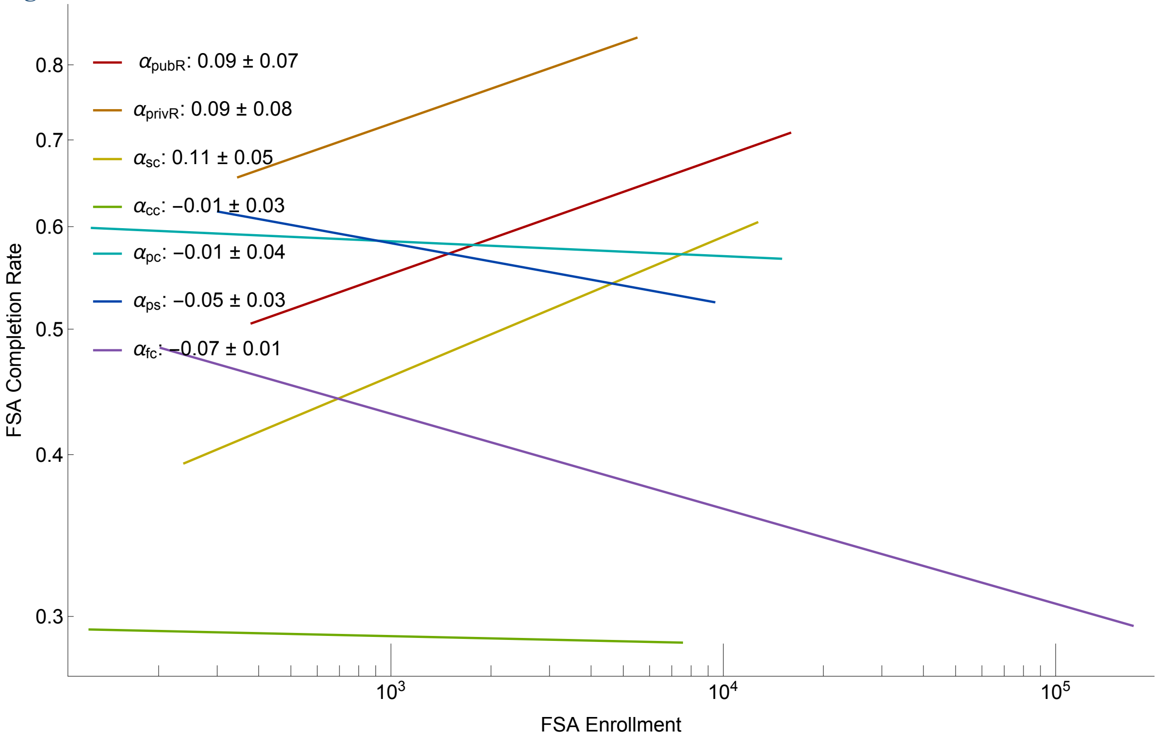

Figure I-12 shows the per capita value of FSA completion rate of all university sectors and the normalized exponent values, versus FSA Cohort as per usual. We see that for all sizes, community colleges have the lowest completion rates and private research universities the highest. In between, the relative performance of sectors depends on the sector. For example, small private non-profit colleges do better than small state colleges, but as these schools get bigger, their performance becomes similar.

Figure I-13

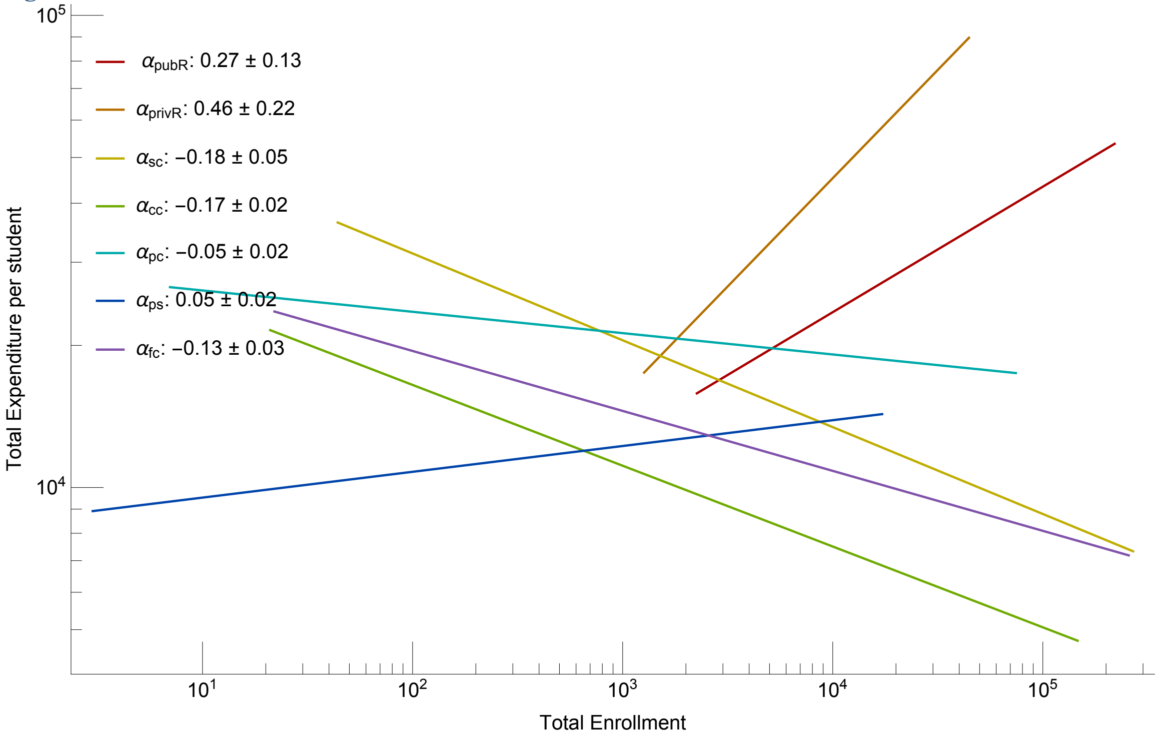

Figure I-13 shows the per capita value of total expenditure per student of all university sectors and the normalized exponent values. There is a drastic contrast between the very high baseline followed by steep increase of expenditure per capita with size of the school in the research universities, with the otherwise lower expenditure per capita, moreover decreasing with size, of schools in the other sectors.

Figure I-14

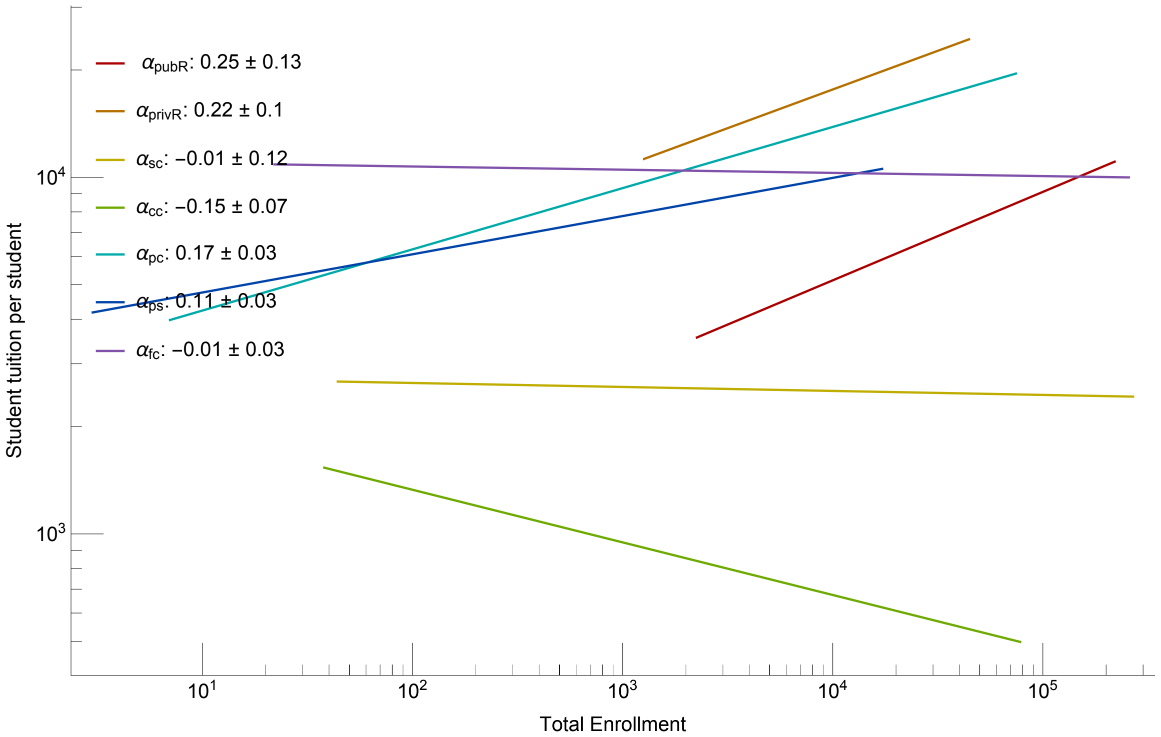

Figure I-14 shows the per capita value of student tuition per student of all university sectors and the normalized exponent values.

Appendix J: Final table of all scaling results

The functional form of the scaling relationships, overall confidence in the scaling parameters, and number of schools considered in each analysis are all useful features for future efforts and provide details not readily available in all of our plots and analyses such as our consideration of the components of revenue and expenditure. Thus we have provided all of the essential scaling relationships from this study in Table J-1.

Table J-1

bleForm=

Variable Name	Public Research Universities	Private Research Universities
FTE	y = **1.01**±0.02x + -0.24±0.18; N:160	y = **1.03**±0.03x + -0.41±0.28; N:102
Full-time undergraduates	y = **0.99**±0.04x + -0.29±0.41; N:159	y = **1.04**±0.11x + -1.09±0.97; N:100
Part-time undergraduates	y = **1.01**±0.2x + -2.37±1.98; N:159	y = **0.71**±0.37x + -0.77±3.4; N:98
Full-time graduate students	y = **1.06**±0.12x + -2.84±1.2; N:160	y = **1.13**±0.16x + -2.58±1.43; N:102
Part-time graduate students	y = **0.83**±0.15x + -0.88±1.52; N:160	y = **0.83**±0.25x + -0.6±2.3; N:100
Student Tuition	y = **1.25**±0.13x + 6.26±1.28; N:159	y = **1.22**±0.1x + 7.78±0.92; N:102
Government Student Grant Aid	y = **0.94**±0.11x + 7.99±1.13; N:159	y = **0.88**±0.21x + 7.98±1.91; N:99
Government Appropriation	y = **1.07**±0.18x + 7.95±1.79; N:157	y = **1.69**±1.24x + -1.11±11.75; N:21
Government Grants and Contracts	y = **1.29**±0.23x + 5.56±2.35; N:159	y = **1.94**±0.49x + -0.71±4.51; N:100
Donation Investment	y = **1.81**±0.31x + -1.24±3.15; N:157	y = **1.69**±0.36x + 2.9±3.32; N:102
Auxiliary	y = **1.59**±0.23x + 2.84±2.35; N:159	y = **1.98**±0.35x + 0.07±3.2; N:102
Total Revenue	y = **1.29**±0.14x + 7.35±1.43; N:159	y = **1.52**±0.24x + 6.15±2.15; N:102
Instruction	y = **1.2**±0.1x + 7.01±1.03; N:159	y = **1.44**±0.18x + 5.53±1.64; N:102
Research	y = **1.52**±0.31x + 2.79±3.1; N:159	y = **1.75**±0.61x + 1.1±5.66; N:84
Public Service	y = **1.74**±0.32x + -0.66±3.24; N:159	y = **1.36**±0.46x + 2.95±4.25; N:61
Maintenance	y = **1.07**±0.11x + 8.01±1.07; N:159	y = **1.33**±0.18x + 6.41±1.65; N:102
Student Service	y = **0.95**±0.12x + 7.6±1.19; N:159	y = **1.09**±0.14x + 7.16±1.28; N:102
Grants and Auxiliary	y = **1.48**±0.2x + 3.94±2.03; N:159	y = **1.83**±0.37x + 1.05±3.35; N:102
Total Expenditure	y = **1.27**±0.13x + 7.62±1.36; N:159	y = **1.46**±0.22x + 6.5±2.04; N:102
Total Faculty	y = **1.16**±0.09x + -3.75±0.91; N:159	y = **1.18**±0.14x + -3.47±1.27; N:102
Instructor Pay	y = **1.2**±0.1x + 6.57±1.; N:159	y = **1.4**±0.17x + 5.45±1.55; N:102
Full-time faculty	y = **1.09**±0.1x + -3.91±1.03; N:159	y = **1.29**±0.18x + -5.35±1.68; N:102
Part-time faculty	y = **1.03**±0.16x + -4.39±1.64; N:159	y = **0.94**±0.17x + -2.66±1.52; N:100
Teaching graduate students	y = **1.43**±0.2x + -8.09±2.; N:155	y = **1.27**±0.4x + -6.69±3.68; N:74
Non-teaching graduate students	y = **1.36**±0.23x + -7.63±2.3; N:149	y = **1.33**±0.37x + -6.99±3.4; N:79
First-time full-time	y = **1.24**±0.06x + -2.47±0.46; N:159	y = **1.17**±0.04x + -1.52±0.27; N:99
FTFT cohort	y = **0.97**±0.08x + -1.83±0.83; N:159	y = **1.07**±0.2x + -3.02±1.84; N:99
FSA Completions	y = **1.09**±0.07x + -1.22±0.57; N:159	y = **1.09**±0.09x + -0.93±0.61; N:94
Net Assets	y = **1.37**±0.19x + 6.66±1.95; N:149	y = **1.68**±0.33x + 5.15±2.98; N:102
All employees	y = **1.18**±0.1x + -3.23±0.99; N:159	y = **1.23**±0.16x + -3.2±1.42; N:102

leForm=

Variable Name	State Colleges	Community Colleges
FTE	y = **0.97**±0.02x + 0.07±0.13; N:382	y = **0.93**±0.01x + 0.08±0.06; N:908
Full-time undergraduates	y = **0.91**±0.04x + 0.32±0.38; N:379	y = **0.86**±0.02x + 0.3±0.13; N:906
Part-time undergraduates	y = **1.08**±0.1x + -2.37±0.86; N:375	y = **1.17**±0.02x + -2.11±0.2; N:885
Full-time graduate students	y = **0.99**±0.16x + -3.4±1.46; N:283	
Part-time graduate students	y = **1.24**±0.15x + -5.05±1.29; N:291	
Student Tuition	y = **0.99**±0.12x + 7.93±1.09; N:347	y = **0.85**±0.07x + 7.87±0.59; N:568
Government Student Grant Aid	y = **0.96**±0.05x + 8.04±0.47; N:377	y = **0.88**±0.02x + 8.55±0.18; N:899
Government Appropriation	y = **0.7**±0.06x + 10.98±0.57; N:368	y = **0.8**±0.03x + 9.87±0.24; N:849
Government Grants and Contracts	y = **0.65**±0.1x + 10.22±0.92; N:377	y = **0.71**±0.05x + 9.19±0.38; N:895
Donation Investment	y = **0.73**±0.18x + 7.4±1.61; N:365	y = **0.83**±0.1x + 4.93±0.82; N:812
Auxiliary	y = **0.7**±0.11x + 10.31±1.; N:377	y = **0.75**±0.06x + 8.38±0.49; N:875
Total Revenue	y = **0.83**±0.05x + 11.08±0.44; N:380	y = **0.81**±0.02x + 10.61±0.14; N:903
Instruction	y = **0.9**±0.04x + 9.44±0.38; N:379	y = **0.81**±0.02x + 9.73±0.14; N:903
Research	y = **0.89**±0.27x + 5.59±2.37; N:302	y = **0.11**±0.3x + 10.46±2.51; N:83
Public Service	y = **0.9**±0.22x + 6.14±1.94; N:337	y = **0.71**±0.14x + 6.91±1.18; N:496
Maintenance	y = **0.8**±0.05x + 10.02±0.45; N:380	y = **0.88**±0.02x + 8.77±0.19; N:903
Student Service	y = **0.78**±0.05x + 9.±0.48; N:380	y = **0.86**±0.03x + 7.8±0.24; N:898
Grants and Auxiliary	y = **0.73**±0.07x + 10.41±0.61; N:379	y = **0.93**±0.05x + 7.97±0.4; N:883
Total Expenditure	y = **0.82**±0.05x + 11.2±0.4; N:380	y = **0.83**±0.02x + 10.5±0.13; N:903
Total Faculty	y = **0.88**±0.04x + -1.67±0.31; N:380	y = **0.84**±0.02x + -1.43±0.17; N:908
Instructor Pay	y = **0.91**±0.04x + 8.91±0.36; N:379	y = **0.82**±0.02x + 9.17±0.14; N:902
Full-time faculty	y = **0.75**±0.04x + -1.21±0.37; N:380	y = **0.67**±0.02x + -1.16±0.17; N:908
Part-time faculty	y = **0.96**±0.08x + -3.42±0.74; N:371	y = **0.97**±0.04x + -3.03±0.34; N:871
Teaching graduate students	y = **1.14**±0.25x + -7.±2.32; N:164	
Non-teaching graduate students	y = **1.**±0.25x + -5.37±2.3; N:190	
First-time full-time	y = **1.11**±0.04x + -1.58±0.27; N:319	y = **0.79**±0.04x + -0.21±0.22; N:890
FTFT cohort	y = **0.93**±0.11x + -1.6±0.96; N:321	y = **0.69**±0.03x + 0.51±0.25; N:890
FSA Completions	y = **1.11**±0.05x + -1.52±0.35; N:353	y = **1.**±0.03x + -1.2±0.21; N:738
Net Assets	y = **0.75**±0.09x + 11.88±0.8; N:360	y = **0.74**±0.04x + 11.21±0.36; N:784
All employees	y = **0.8**±0.04x + -0.17±0.32; N:381	y = **0.77**±0.02x + -0.24±0.15; N:908

leForm=

Variable Name	Non-profit Private Colleges	Professional Schools
FTE	y = **1.**±0.01x + -0.19±0.05; N:1373	y = **1.01**±0.01x + -0.17±0.03; N:2230
Full-time undergraduates	y = **1.**±0.03x + -0.5±0.19; N:1162	y = **1.01**±0.01x + -0.27±0.07; N:2205
Part-time undergraduates	y = **0.85**±0.07x + -1.46±0.47; N:1059	y = **0.87**±0.05x + -0.76±0.27; N:1102
Full-time graduate students	y = **0.55**±0.06x + 0.82±0.43; N:991	
Part-time graduate students	y = **0.77**±0.07x + -0.84±0.51; N:916	
Student Tuition	y = **1.17**±0.03x + 7.96±0.22; N:1317	y = **1.11**±0.03x + 8.22±0.18; N:1975
Government Student Grant Aid	y = **0.98**±0.04x + 7.61±0.3; N:1179	y = **0.99**±0.02x + 8.34±0.1; N:2122
Government Appropriation	y = **0.45**±0.24x + 9.33±1.78; N:119	
Government Grants and Contracts	y = **0.85**±0.08x + 7.08±0.56; N:993	
Donation Investment	y = **0.82**±0.06x + 9.61±0.41; N:1333	y = **0.98**±0.16x + 3.18±0.87; N:517
Auxiliary	y = **1.18**±0.06x + 6.77±0.39; N:1305	y = **0.51**±0.07x + 9.37±0.34; N:1573
Total Revenue	y = **0.99**±0.03x + 10.18±0.18; N:1358	y = **1.03**±0.02x + 9.22±0.12; N:2141
Instruction	y = **0.99**±0.02x + 8.85±0.17; N:1356	y = **0.97**±0.03x + 8.44±0.13; N:2140
Research	y = **0.65**±0.14x + 8.21±1.04; N:338	
Public Service	y = **0.69**±0.14x + 7.8±1.02; N:413	
Maintenance	y = **0.89**±0.02x + 9.66±0.17; N:1354	
Student Service	y = **1.16**±0.03x + 6.71±0.23; N:1328	y = **1.17**±0.04x + 6.31±0.23; N:2014
Grants and Auxiliary	y = **1.05**±0.07x + 7.47±0.49; N:1233	y = **0.71**±0.13x + 8.47±0.68; N:1129
Total Expenditure	y = **0.95**±0.02x + 10.28±0.15; N:1358	y = **1.06**±0.02x + 8.99±0.13; N:2141
Total Faculty	y = **0.89**±0.02x + -1.49±0.14; N:1362	y = **0.76**±0.02x + -1.35±0.1; N:2221
Instructor Pay	y = **0.98**±0.02x + 8.52±0.16; N:1355	
Full-time faculty	y = **0.88**±0.02x + -2.21±0.16; N:1347	y = **0.69**±0.02x + -1.57±0.12; N:2086
Part-time faculty	y = **0.82**±0.04x + -1.85±0.28; N:1215	y = **0.63**±0.04x + -1.46±0.23; N:1654
Teaching graduate students	y = **0.48**±0.15x + -1.29±1.11; N:167	
Non-teaching graduate students	y = **0.4**±0.13x + -0.73±0.96; N:285	
First-time full-time	y = **1.09**±0.02x + -1.15±0.11; N:1035	y = **1.02**±0.02x + -0.64±0.1; N:730
FTFT cohort	y = **0.98**±0.04x + -1.93±0.32; N:1049	y = **0.95**±0.06x + -0.53±0.32; N:735
FSA Completions	y = **0.99**±0.04x + -0.47±0.27; N:615	y = **0.96**±0.06x + -0.26±0.42; N:168
Net Assets	y = **1.01**±0.05x + 10.49±0.34; N:1305	
All employees	y = **0.88**±0.02x + -0.62±0.13; N:1370	y = **0.8**±0.02x + -0.9±0.1; N:2227

leForm=

Variable Name	For-profit Colleges
FTE	y = **0.98**±0.01x + -0.13±0.09; N:647
Full-time undergraduates	y = **0.96**±0.05x + -0.4±0.35; N:614
Part-time undergraduates	y = **0.98**±0.06x + -1.12±0.39; N:470
Full-time graduate students	y = **0.68**±0.12x + -0.41±0.81; N:254
Part-time graduate students	y = **0.83**±0.15x + -1.13±1.06; N:169
Student Tuition	y = **0.99**±0.03x + 9.32±0.21; N:608
Government Student Grant Aid	y = **0.97**±0.05x + 8.52±0.34; N:589
Government Appropriation	
Government Grants and Contracts	
Donation Investment	y = **0.96**±0.21x + 3.05±1.46; N:163
Auxiliary	y = **0.93**±0.14x + 6.7±0.94; N:383
Total Revenue	y = **1.**±0.03x + 9.69±0.19; N:614
Instruction	y = **0.93**±0.04x + 8.59±0.29; N:615
Research	
Public Service	
Maintenance	y = **0.75**±0.15x + 10.58±0.89; N:17
Student Service	y = **0.85**±0.07x + 8.63±0.46; N:613
Grants and Auxiliary	y = **1.2**±0.3x + 4.34±2.01; N:395
Total Expenditure	y = **0.87**±0.03x + 10.46±0.17; N:615
Total Faculty	y = **0.83**±0.04x + -1.22±0.24; N:646
Instructor Pay	y = **0.92**±0.24x + 8.6±1.47; N:17
Full-time faculty	y = **0.79**±0.05x + -2.63±0.31; N:613
Part-time faculty	y = **0.77**±0.05x + -1.06±0.31; N:623
Teaching graduate students	y = **0.21**±0.31x + 0.55±2.1; N:21
Non-teaching graduate students	
First-time full-time	y = **0.96**±0.05x + -1.18±0.21; N:281
FTFT cohort	y = **0.62**±0.15x + -0.7±1.01; N:317
FSA Completions	y = **1.06**±0.09x + -1.23±0.67; N:82
Net Assets	
All employees	y = **0.8**±0.03x + -0.43±0.17; N:646

Appendix K: References

Bettencourt, L. (2013). The Origins of Scaling in Cities. *Science*, 340(6139), pp.1438-1441.

Bok, D. (2013). *Higher Education in America*. Princeton University Press.

Carnegie Foundation for the Advancement of Teaching (2011). *The Carnegie Classification of Institutions of Higher Education*, 2010 edition, Stanford, CA: Author. Retrieved July 12, 2018 from http://carnegieclassifications.iu.edu/

Chetty, R., Friedman, J. N., Saez, E., Turner, N., & Yagan, D. (2017). *Mobility Report Cards: The Role of Colleges in Intergenerational Mobility* (No. w23618). National Bureau of Economic Research.

Crow, M.M., Dabars, W.B. (2015). *Designing the New American University*. JHU Press.

Delta Cost Project (2011). *Delta Cost Project Documentation of IPEDS Database and Related Products. Delta Cost Project. (2011).* Washington, DC: Delta Cost Project. Retrieved October 28, 2018 from https://deltacostproject.org/sites/default/files/database/DCP_History_Documentation.pdf

Desrochers, D.M., and Sun, J. (2015). *IPEDS Analytics: Delta Cost Project Database 1987-2012* (NCES 2015-091). U.S. Department of Education. Washington, DC: National Center for Education Statistics. Retrieved March 25, 2018 from http://nces.ed.gov/ipeds/deltacostproject/download/DCP_Data_File_Documentation_1987_2012.pdf

Financial Reporting in Higher Education. (2014). Hanover Research. Washington, DC. Retrieved June 29, 2018 from https://www.hanoverresearch.com/media/Financial-Reporting-in-Higher-Education.pdf

Ginder, S.A., Kelly-Reid, J.E., and Mann, F.B. (2014). *2013-14 Integrated Postsecondary Education Data System (IPEDS) Methodology Report* (NCES 2014-067). U.S. Department of Education. Washington, DC: National Center for Education Statistics. Retrieved March 25, 2018 from http://nces.ed.gov/pubs2014/2014067.pdf

Goldstein, L. and Menditto, S. (2017). *GASB and FASB*. NACUBO. National Association of College and Business University Offices.

Hurlburt, S., Peek, A., and Sun, J. (2017). *Delta Cost Project Database 1987-2015: Datafile Documentation*. Delta Cost Project at American Institutes for Research. Retrieved March 25, 2018 from http://deltacostproject.org/sites/default/files/database/DCP_Database_Documentation_1987-2015.pdf

Institutional Eligibility Under the Higher Education Act of 1965, As Amended. 34 CFR 600.4, 600.5, and 600.6, Apr. 5 1988. http://ifap.ed.gov/fsahandbook/attachments/0910FSAHbkVol2Ch1School.pdf http://www.gpo.gov/fdsys/pkg/CFR-2011-title34-vol3/pdf/CFR-2011-title34-vol3-part600.pdf

Jaquette, O. and Parra, E. (2016) The problem with the delta cost project database. *Research in Higher Education*, 57(5):630–651. doi: 10.1007/s11162-015-9399-2

U.S. Department of Education (2018). *Data Documentation for College Scorecard* College Scorecard Project, U.S. Department of Education. Retrieved April 18, 2018 from https://collegescorecard.ed.gov/assets/FullDataDocumentation.pdf

Using Federal Data to Measure and Improve the Performance of U.S. Institutions of Higher Education. (2015). Executive Office of the President of the United States: Council of Economic Advisors. Retrieved April 22, 2018 from https://collegescorecard.ed.gov/assets/UsingFederalDataToMeasureAndImprovePerformance.pdf
